# Significant reduction of corrosion of stainless steel by strong-field laser surface passivation

**DOI:** 10.1038/s41377-025-01952-5

**Published:** 2025-10-05

**Authors:** Liansheng Zheng, Hongwei Zang, Yuan Liu, Yukun Xiao, Yingbo Cong, Zhen Cheng, Ganwen Chen, Zhenxiang Xing, Jisheng Pan, Qing Jiang, Wei Chen, Kaoru Yamanouchi, Huailiang Xu, Ruxin Li

**Affiliations:** 1 State Key Laboratory of Integrated Optoelectronics, College of Electronic Science and Engineering, Jilin University, Changchun, China; 2 State Key Laboratory of High Field Laser Physics, Shanghai Institute of Optics and Fine Mechanics, Chinese Academy of Sciences, Shanghai, China; 3 Department of Chemistry, National University of Singapore, Singapore, Singapore; 4 Joint School of National University of Singapore and Tianjin University, International Campus of Tianjin University, Fuzhou, China; 5 School of Mechanical and Aerospace Engineering, Jilin University, Changchun, China; 6 State Key Laboratory of Luminescence and Applications, Changchun Institute of Optics, Fine Mechanics and Physics, Chinese Academy of Sciences, Changchun, China; 7 Institute of Materials Research and Engineering, Agency for Science, Technology and Research A*STAR, Singapore, Singapore; 8 School of Materials Science and Engineering, Jilin University, Changchun, China; 9 Institute for Attosecond Laser Facility, The University of Tokyo, Tokyo, Japan; 10 School of Optoelectronic Engineering, Xidian University, Xi’an, China; 11 School of Physical Science and Technology, Shanghai Tech University, Shanghai, China

**Keywords:** Laser material processing, Nonlinear optics

## Abstract

Stainless steels are basic corrosion-resistant materials, but despite great efforts for over a century, they still suffer inevitably from environmental erosions by ubiquitous chemical reactions, resulting in typical corrosion rates at dozens of μm∙yr^-1^. Here, we developed a strong-field laser passivation strategy to obtain super corrosion-resistant stainless steels through forming a hybrid µm-Fe_3_O_4_/Fe_2_O_3_/Cr_2_O_3_ passivation layer with unique bionic taro-leaf-like hierarchically heterogeneous Cassie-state micro/nanostructure morphologies. We observed up to 100,000-fold reduction in the corrosion rate of AISI 304 steel in saline, acidic as well as alkaline solutions. The ultralow corrosion rate can remain for >6500 hours. The generality was exemplified by exhibiting extreme anticorrosion enhancements of AISI 316, 420, 201, 430, and 2205 steels under the same conditions. This study reveals a new strategy for achieving super corrosion-resistant performance of stainless steels in various harsh environments.

## Abstract (web summary)

Strong-field laser surface passivation achieves orders of magnitude reduction in the corrosion rate of stainless steels.

## Graphical abstract

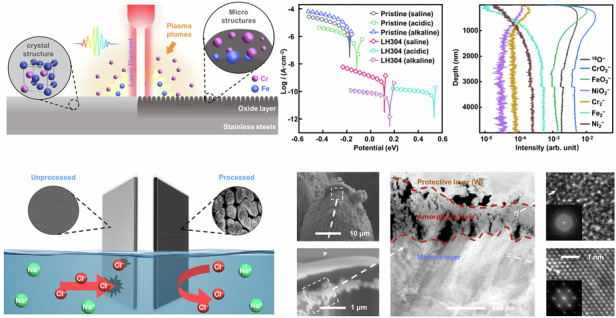

## Introduction

Steels are widely used not only in daily life but also in urban infrastructure and industry owing to their good ductility, thermal and electrical conductivity, weldability, and malleability. However, the corrosion of steels by chemical reactions under aggressive environments such as humid marine atmosphere, saline seawater, and acidic/alkaline electrolytes results in massive annual losses worldwide^1–5^. The most often used anticorrosion strategy is to form corrosion-resistant polycrystalline stainless steels by the addition of alloying elements to steels, typically Ni and Cr. It has been known that Cr in the stainless-steel alloys plays a key role in creating a thin passive oxide layer against oxidation^6,7^, making the steels essentially rust-proof with a common corrosion rate of a few to hundreds of μm·yr^−1^ under most corrosive environments^1^. Because of this anti-corrosion mechanism, stainless steels can retain their original appearance for long periods under normal conditions and survive for a longer period than most other metals. Nevertheless, stainless steels often show a sudden onset of corrosion with a sharp rise in the corrosion rate due to localized pitting corrosion that happens with only small changes in conditions such as temperature, potential, or solution concentrations^8,9^. This type of sudden corrosion leads to serious problems in stainless-steel-based facilities such as underground sewer infrastructures, port terminals, and marine oil and gas exploiting facilities^10–13^, whose lifetimes can be significantly shortened by the seriously rusted stainless steels.

To control and prevent the pitting corrosion, a variety of technologies for coating stainless-steel surfaces have been developed^14–16^. However, the passive coating films made by organic, inorganic, or carbon-based materials as corrosion inhibitors (for example, polymer^14^, metallic oxide^15^, and graphite^16^) are not completely impermeable to certain corrosive species such as chlorine ions and hydroxyl ions when the surfaces are exposed chronically to harsh environments. In addition, microscopic pores and cracks frequently created in coating films may even accelerate local corrosions, resulting in deterioration of the corrosion resistance of stainless steels^17^. On the other hand, laser processing of stainless steels shows an excellent anti-pitting property through the formation of micro/nano-structured hydrophobic surfaces^18^, which creates a physical barrier between the metal surface and electrolyte^19,20^. Unfortunately, this technique can improve the anticorrosion performance of stainless steels only by one to two orders of magnitude so far^17–28^.

Here, we provide a strategy for achieving an ultrahigh anticorrosion performance of stainless steels via strong-field laser processing using far-field femtosecond laser pulses in the filamentation regime, which can be straightforwardly extended to the large-scale processing of rough and irregular stainless-steel surfaces at a standoff distance^29^. We find unexpectedly that the corrosion resistance of various types of stainless steels (AISI 304, 316, 420, 201, 430, and 2205) in saline, acidic, and alkaline solutions is enhanced significantly by the strong-field laser filament (SLF) processing. In the case of AISI 304 stainless steel, we find that the SLF processing not only suppresses the corrosion rates by as much as 4−5 orders of magnitude but also remarkably increases the durability. Furthermore, we reveal that the SLF processing promotes not only the formation of a passive hybrid Fe_3_O_4_/Fe_2_O_3_ and Cr_2_O_3_ layer having abundant Cr content^30^, which reinforces the surface oxidation resistance, but also the formation of unique bionic taro-leaf-like hierarchically heterogeneous Cassie-state micro/nanostructures having deep ravines and flat mountaintops. Indeed, the micro/nanostructures greatly suppress pitting corrosion and build up a physical barrier through ultra-hydrophobicity to inhibit an exposure of the metal surfaces to corrosive solutions.

## Results

### Surface engineering strategy and anticorrosion performance

**Fig. 1:**
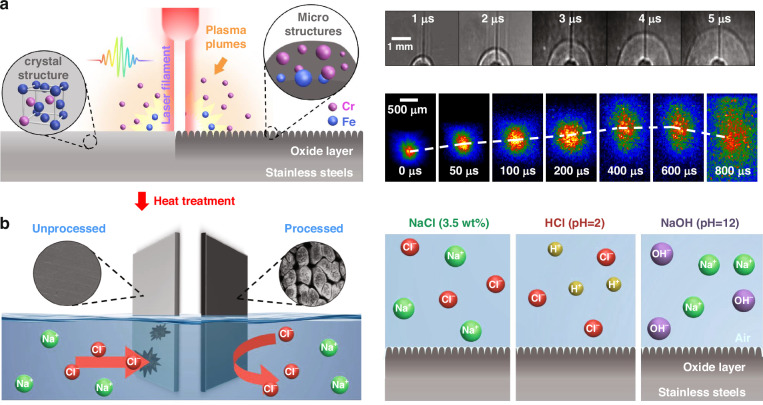
Engineering strategy for super corrosion-resistance stainless steels. **a** Schematic of the SLF processing of stainless steel (left), and the images for laser filament-induced shock wave (right top) and plasma plume (right bottom); **b** Conceptual steel corrosion occurring in a saline solution with the unprocessed and processed steel surfaces (left), and schematic of composite passivation layers for anticorrosion in saline (NaCl), acidic (HCl) and alkaline (NaOH) solutions (right)

Conventionally, laser processing of metal surface to achieve metal anticorrosion has been conducted in a near-field manner, that is, microstructures are created on the metal surface by tight focusing of laser light at the surface to make the metal surface water-resistant^19,21,22,24^. However, this approach has a drawback that the original surface passivation layer may be ruined, leading water-resistant surface to be the dominant origin for the 1−2 orders of magnitude reduction in the corrosion rates^17–28^. In the present study, we adopt a one-step-forming strategy (see Fig. 1 and Fig. S1) that is entirely different from the near-field approach. We fabricate stainless steels by a far-field SLF processing using a long and thin femtosecond-laser filament, providing a constant laser field intensity as high as 50−100 (TW·cm^−2^)^31,32^, which generates a swelling shock wave interfering with air laser filament followed by a luminescing plasma plume from the target (Fig. 1a). We show that this SLF processing introduces bionic taro-leaf-like hierarchically heterogeneous micro/nanostructures on the steel surface and transports Cr atoms, *i.e*., the lightest metal atoms, from the bulk domain of the stainless steel to the surface to form a hybrid µm-Fe_3_O_4_/Fe_2_O_3_/Cr_2_O_3_ passivation layer with more abundant Cr concentration. After the SLF processing, we heat the stainless steel samples at 150 °C to reduce the surface energy as well as to form an air shield on the ultrahydrophobic steel surface. By the SLF processing with the low-temperature heating treatment, we can produce composite protective layers on the stainless-steel surface, which significantly suppress the metal-electrolyte surface reactions in harsh saline, acidic as well as alkaline aqueous solution environments (Fig. 1b) (for more details of the experimental procedures, see Methods).

### Anticorrosion performance

**Fig. 2:**
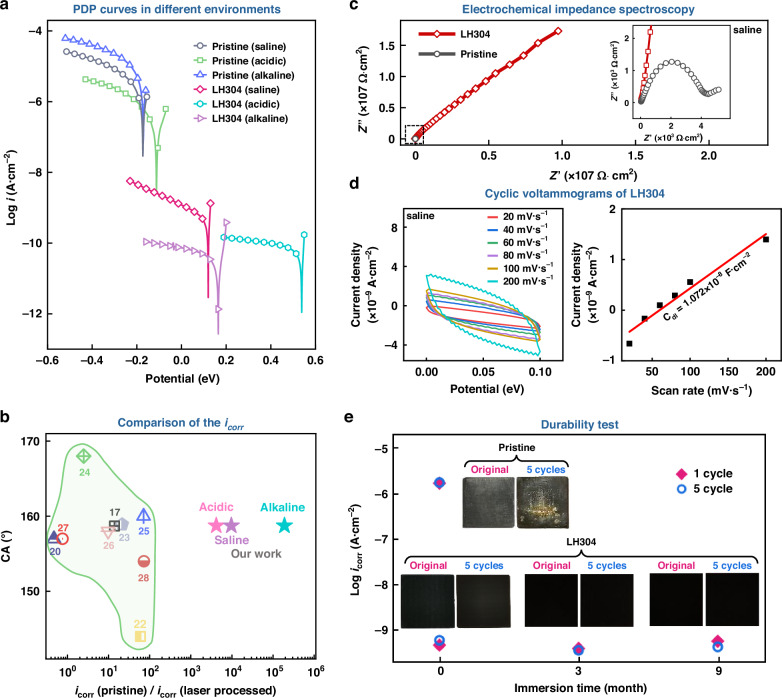
Corrosion characteristics of the stainless steels. **a** Cathodic PDP curves of the pristine and LH304 samples measured in saline, acidic, and alkaline solutions; **b** The *i*_corr_ and contact angle (CA) values obtained in our work (LH304) and those (see Table S2) reported in literatures by laser processing techniques; **c** Nyquist plots obtained from EIS for the pristine and LH304 samples. Inset: Zoomed-in Nyquist plots; **d** Cyclic voltammogram (left) and the measured (rectangle dots) and fitted (solid line) double layer charging current densities versus the scan rate (right) of LH304; **e** Corrosion current densities of the pristine and LH304 samples immersed in the 3.5 wt.% NaCl aqueous solution for nine months (>6500 h) by one-cycle and five-cycle PDP measurements. Inset: Surface appearances taken by a digital camera for pristine and LH304 without (original) and with (5 cycles) PDP measurements

Electrochemical measurements were first carried out to evaluate the anticorrosion performance of the processed 304 stainless-steel samples, which are called hereafter LH304. Potentiodynamic polarization (PDP) measurements of the pristine (AISI 304) and processed (LH304) stainless-steel samples in three types of corrosive environments, i.e., saline (3.5 wt.% NaCl), acidic (HCl, pH=2), and alkaline (NaOH, pH = 12) aqueous solutions yield the cathodic (Fig. 2a) and anodic (Fig. S2a) polarization curves. In Fig. 2a and Fig. S2a, the corrosion current density *i*_corr_ in the ordinate represents the rate of corrosion occurring at the working electrode in terms of the current per unit area^33,34^ and the corrosion potential *E*_corr_ in the abscissa represents the corrosion tendency^17^. From the cathodic (Fig. 2a) and anodic (Fig. S2a) curves of the LH304 surfaces, the corrosion parameters, *E*_corr_, *i*_corr_, and *CR* (corrosion rate), are obtained as listed in Table S1, from which it can be noted that the *i*_corr_ values for LH304 obtained under all the three corrosive environments are smaller than those for the untreated pristine surface by 4−5 orders of magnitude. The significantly reduced corrosion rates achieved in the present study are even smaller by about 2−3 orders of magnitude than those obtained using the state-of-the-art anticorrosion techniques (Fig. 2b, Table S2). Moreover, the *E*_corr_ values of the pristine sample shift to the positive values of LH304, which shows that the LH304 surfaces are more difficult to be corroded than the pristine 304 surfaces. The reproducibility for the improved anticorrosion performance is also verified by performing the PDP tests of different fabricated steel samples (see Fig. S3 and Table S3). In order to confirm that the significant improvement of the anticorrosion performance is achieved by the SLF processing with the low-temperature heating treatment, we performed the PDP measurements of the pristine sample processed only with the heating treatment, which is referred to as H304. As seen in Fig. S2b and Table S1, *i*_corr_ = 2.082 × 10^-6^ A·cm^-2^ of H304 is slightly lower than that of the pristine sample, while *E*_corr_ remains nearly unchanged, indicating that the anticorrosion performance of the H304 surface is improved only slightly. It is therefore confirmed that, to give the super-corrosion resistance to AISI 304 stainless steel, the SLF processing is a prerequisite.

To further verify the anticorrosion performance of LH304, we carried out electrochemical impedance spectroscopy (EIS) analyses (Fig. 2c, Fig. S4, and Table S4) of LH304 in the 3.5 wt.% NaCl aqueous solution. In the Nyquist plots (Fig. 2c) obtained from EIS, the capacitive loop with a larger diameter represents a higher corrosion resistance with a lower corrosion current density^17^. The Nyquist plots show that the diameter of the capacitive loop of LH304 is much larger than that of the pristine surface (see the inset), which is consistent with the corrosion resistance obtained in the PDP measurements shown in Fig. 2a. In addition, we employed the equivalent circuit diagrams (Fig. S4a, b) to fit the EIS results (see Table S4 for the detailed fitting results). The charge transfer resistance *R*_ct_ of LH304 is three orders of magnitude higher than that of the pristine surface, and the value of *CPE*_dl_ (Constant phase element associated with the electric double-layer capacitor in the substrate and film) is three orders of magnitude lower than that of the pristine surface. These results indicate that it is much more difficult for Cl^−^ to penetrate the passivation layer^17,35^, that is, the contact probability between Cl^−^ and the steel matrix becomes smaller, leading to the higher corrosion resistance.

The improved anticorrosion performance of the processed LH304 surface can also be seen from the Bode plots, which depict the variations of the impedance modulus and phase angle with frequency. We measured the Bode plots of the pristine and LH304 samples (Fig. S4c, d). Generally, high and stable phase angle maxima across a wide frequency range and large absolute impedance values|Z| represent better anticorrosion performance^35^. The Bode angle plots (Fig. S4c) show that the LH304 surface has the largest phase angles in the entire frequency range than the pristine surface and that the phase angles of the LH304 surface take the maximum value of 74−76° in the wide frequency range of 10^0^−10^3^ Hz. Besides, in the Bode impedance plots Fig. S4d), the absolute impedance values|*Z*|, reflecting the diameters of the capacitive loops, show that the LH304 surface takes larger |*Z*| values than the pristine surface in the entire frequency range and that the difference becomes significantly larger in the low-frequency range. Furthermore, the results of our measurements of cyclic voltammetry (CV) (Fig. 2d and Fig. S4e and 4f) show that the electric double-layer capacitor for the LH304 surface, *C*_dl_ = 1.072 × 10^−8^ F·cm^−2^, is smaller than that for the pristine surface, *C*_dl_ = 1.146 × 10^−5^ F·cm^−2^, by three orders of magnitude. Because the electric double-layer capacitor directly reflects the electrochemically active surface area (ECSA)^36^, the significant reduction of the *C*_dl_ values means that the LH304 surface is much more resistant to the corrosive environments than the pristine surface.

To examine the durability of the anticorrosion performance of LH304, we immersed the LH304 samples into a 3.5 wt.% NaCl aqueous solution for three months (>2100 h) and the other LH304 samples for nine months (>6500 h). We then recorded the PDP curves for the two sets of the LH304 samples (Fig. 2e and Fig. S5). The corresponding corrosion parameters listed in Table S5, Fig. 2e and Fig. S5 show that the anticorrosion performances of LH304 are kept unchanged even after the nine-month immersion into the saline solution. Furthermore, we conducted the in-situ PDP measurements of the same sample five times consecutively to further examine the durability of the samples. After 5-cycle PDP measurements, the pristine sample exhibits severe corrosion traces on the surface, which is consistent with our understanding that the PDP measurements are, in general, destructive^37^. On the other hand, as shown by the photos in the insets of Fig. 2e, the LH304 samples retain the original appearances with *i*_corr_ keeping the values of ∼5.0 × 10^−10^ A·cm^−2^, confirming clearly the durability of the super-anticorrosion performance of LH304.

To examine the mechanical stability of LH304, we performed the friction coefficient measurement, as well as a series mechanical wear tests including simulated rainfall, sandpaper abrasion, external compression, wave-simulated vibration, and ultrasonic cleaning. The results in Fig. S6 demonstrate that the processed samples possess more mechanical robustness than that of the pristine sample. Moreover, to quantify the Arrhenius-type relationship between the solution thermodynamic parameters and corrosion mechanisms, a temperature-controlled electrochemical measurement was performed at 20 °C, 30 °C, 40 °C, 50 °C and 60 °C in the 3.5 wt.% NaCl aqueous solution. Based on the resultant PDP curves of LH304 (Fig. S7 and Table S6), we obtained the activation energy of LH304 to be >50 kJ∙mol^-1^, which is much larger than the value (28.42 kJ∙mol^-1^) of the pristine 304 steel^38^. This high activation energy further demonstrates the excellent corrosion resistance of the processed samples.

## Discussion

### Mechanisms of corrosion resistance

**Fig. 3:**
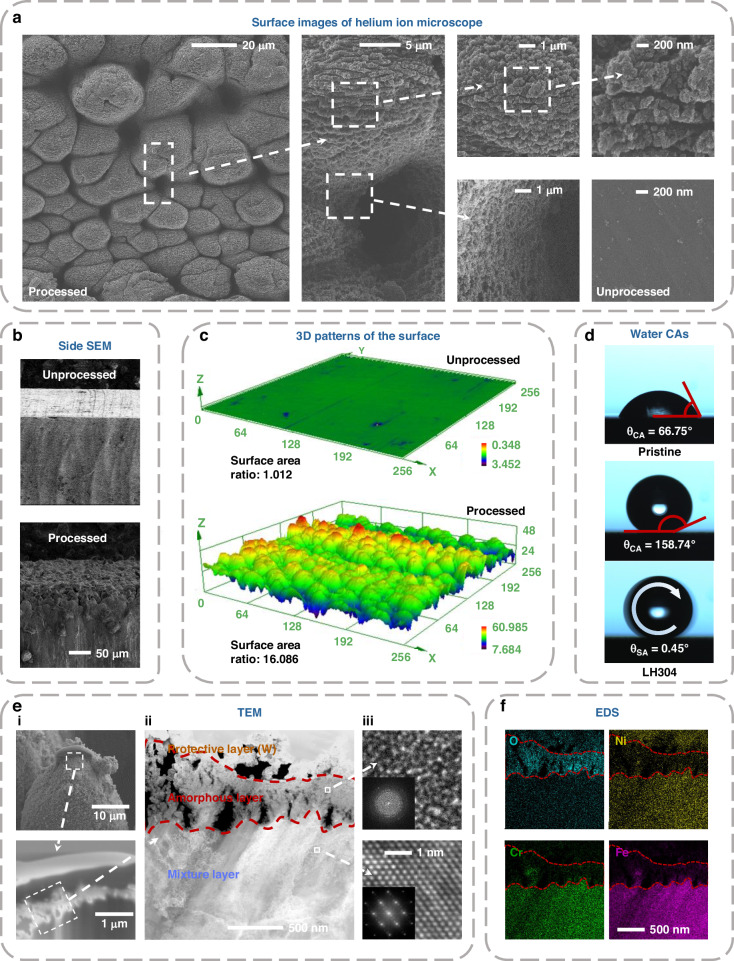
Morphology, wettability and crystalline phase of LH304 stainless steel surfaces. **a** Surface images obtained by helium-ion microscope (HIM) with different magnifications; **b** Side-view SEM images; **c** 3D patterns of the stainless-steel samples before and after laser filament processing; **d** CA and RA results; **e** Images of (**e**_**i**_) the LH304 sample prepared by FIB, (**e**_**ii**_) dark-field TEM, and (**e**_**iii**_) HRTEM (Inset: fast Fourier transform (FFT) pattern). **f** EDS of O, Ni, Fe and Cr elements

To explore the origin of the super-anticorrosion performance, we first examine the morphologies of the processed 304 steel surfaces using a Helium-ion microscope and a field emission scanning electron microscope (SEM). In the recorded surface-topography images (Fig. 3a, b and Fig. S8), we can see unique taro-leaf-like and hierarchically heterogeneous micro- and submicro-structures with flat mountaintops, which exhibit a marked difference from regular spike-like structures created by near-field laser processing^22^. In addition, we find the finer sub-microstructures having grain-like features are created in the flat-top plateau area (see Fig. S9). The formation of the hierarchical structures is due to the unique energy distribution of the filament, where a high-intensity filament core surrounded by a weak-intensity energy reservoir. The 3D patterns (Fig. 3c) clearly show that the sub-microstructures give a significant increase in the surface area ratio. These hierarchical sub-micro and micro-structures efficiently suppress the wettability of the stainless-steel surfaces (Fig. 3d and S10). The CA and rolling angle (RA) measurements reveal that the laser processing first induces an ultrahydrophilic surface at the Wenzel state, where the water droplet is fully sunk into the gaps among the microstructures on the sample surface without the heat treatment, which is hereafter called L304. After the heat treatment that lowers the surface energy^17^, super-hydrophobicity is realized on the LH304 surface in the Cassie state, having the high water-CA of 158.74°, and the low RA of 0.45°. The ultra-hydrophobic property benefits the formation of an additional air layer on the steel surface, which plays a role of an inherent insulator and impedes direct contact between the corrosive media and the stainless-steel surface.

To further investigate the strong-field laser-induced changes in the chemical compositions and crystalline phases of the metal surface, we employed a focused ion beam (FIB) to dig a small piece of the LH304 sample with a size of ∼0.1×3×4 μm^3^ (Fig. 3e(i)) and performed the transmission electron microscope (TEM) measurement (Fig. 3e(ii)). The dark-field TEM image and the high-resolution (HRTEM) images (Fig. 3e(iii)) reveal that the sample surface is composed of a thin amorphous layer of about 500 nm and a thick crystalline/amorphous mixture layer of a few μm (Fig. 3e(ii)) (More HRTEM images can be found in Fig. S11), where the top protective tungsten layer was introduced by the FIB technology. The crystalline phase is dominated by the austenitic structure, which is consistent with the XRD results (Fig. S12 and S13a). The energy dispersive spectroscopy (EDS) results (Fig. 3f and Fig. S13c) with the measured area corresponding to the dark field TEM image show that there is rich oxygen content doped into both the amorphous layer and the crystalline/amorphous mixture layer, indicating the formation of the oxide passivation layer. Furthermore, the TEM, HRTEM, and EDS measurements of L304 (Fig. S13b and d, e) result in almost the same results as those of LH304, indicating that the low-temperature heat treatment has negligible effects on the chemical compositions and crystalline phases of the laser processed metal surface.

**Fig. 4:**
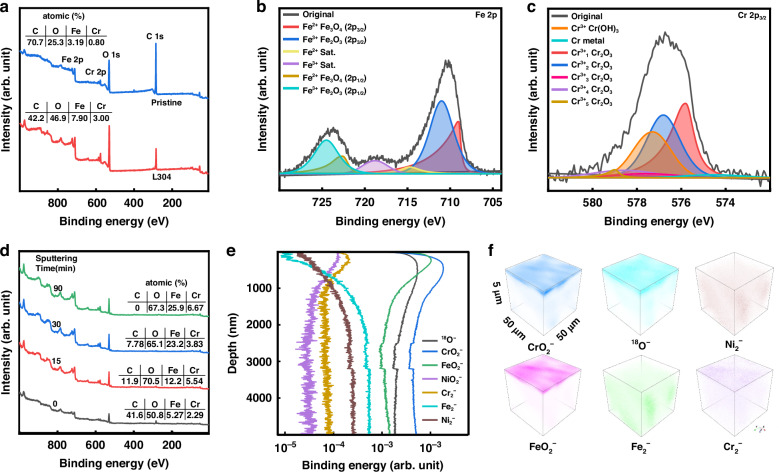
Surface element contents of L304 steels. **a** XPS spectra; **b** XPS high-resolution Fe 2p spectrum; **c** XPS high-resolution Cr 2p spectrum; **d** XPS depth profiles obtained from the sputtering time of 0, 15, 30, 90 min, respectively; **e** ToF- SIMS depth profiles. **f** 3D ToF–SIMS image of CrO_2_^−^, FeO_2_^−^, ^18^O^−^, Fe_2_^−^, Ni_2_^−^ and Cr_2_^−^

Besides the morphological changes on the stainless-steel surfaces, the laser treatment also induces discernible modifications in the chemical compositions (Fig. 3f). To further investigate the change in the chemical compositions, we performed X-ray photoelectron spectroscopy (XPS) measurements. As shown in Fig. 4, the XPS results of L304 show that the laser processing increases the relative oxygen concentration on the surface from 25.3% (pristine) to 46.9% (Fig. 4a). The laser filament impact on steel alloy produces a plasma plume (Fig. 1a) composed mainly of neutral metal atoms, metal atom cations (Fig. S14a and S14b), and a variety of active oxygenous species such as O, O_2_^+^, and O_3_ from the air filament^31,32^. The interaction of the oxygenous products with the readily oxidizable Fe and Cr contents in the plasma promotes the formation of oxide products. Indeed, the analysis of the Fe 2p spectral peak structure of the sample (Fig. 4b) reveals that the Fe_2_O_3_ peaks can be found at 711.0 eV and 724.5 eV in addition to the Fe_3_O_4_ peaks at 709.0 eV and 722.6 eV^21,39^, showing that the hybrid Fe_3_O_4_/Fe_2_O_3_ oxide layer is created by the laser processing. On the other hand, the analysis of the Cr 2p spectral peak structure at 572–582 eV (Fig. 4c) shows that Cr_2_O_3_ is also created at the surfaces. In addition, the laser processing lowers significantly the relative carbon concentration on the stainless-steel surface from 70.7% (pristine) to 42.2%. The carbon atoms in the laser filament-induced plasma can be oxidized to be gaseous CO or CO_2_, which eventually escape from the stainless-steel surface. The super-anticorrosion surface is also examined using ultraviolet photoelectron spectroscopy (UPS), which can provide information on the density of states of the valence band of the laser-processed sample at each sputtered depth. The UPS results (Fig. S14c) show that throughout the sputtering process, the valence band maximum (VBM) gradually lowers to the Fermi level. This shift with the higher VBM at the surface confirms that the laser-processed surface is less susceptible to oxidation.

The variation in the chemical composition of Fe, Cr, C, and O obtained from the XPS survey scans for the L304 steel is shown in Fig. 4d. The high oxygen concentration throughout the sputtered depths originates from the filament-induced oxide layer. Moreover, the Cr/Fe ratios at the sputtering times of 0 min and 15 min take almost the same value of ~0.43, decrease to ~0.17 at the sputtering time of 30 min, and increase to ~0.26 at the sputtering time of 90 min, which is close to the Cr/Fe ratio (=0.25) of pristine samples. This observation implies that the strong-field filament processing promotes the transport of Cr atoms to the surface, which contributes to the improvement of the corrosion resistance^21^. The transport of Cr atoms to the surface may be ascribed to their low specific density, which makes the Cr atoms have a higher speed to leave the surface during the laser-filament induced ablation, and then, come back to the surface later than other elements. Furthermore, the higher melting point of Cr than that of Ni and Fe may lead to a smaller amount of Cr atoms to be ablated from the surface.

To precisely determine the atomic and molecular components on the stainless-steel surface at different depths, we carry out time-of-flight (ToF)-secondary ion mass spectrometry (SIMS) measurements of L304. The analyses of the ToF-SIMS data of the processed sample show the depth profiles of negative ions: ^18^O^−^, CrO_2_^−^, FeO_2_^−^, NiO_2_^−^, Cr_2_^−^, Fe_2_^−^ and Ni_2_^−^ (Fig. 4e, f). The high intensities of the signals of ^18^O^−^, CrO_2_^−^, FeO_2_^−^, and NiO_2_^−^ reflect the enrichment of the metallic oxides. Based on the depth profiles, the metal/oxide interface is estimated to be positioned at the depth where the intensities of the metallic signals of Ni_2_^−^, and Fe_2_^−^ takes 80% of their maximum intensities^40^, and thus, the thickness of the oxide layer is estimated to be about 1.6 µm, which is much thicker than the typical thickness (1−3 nm) of a native surface oxide layer of AISI 304 stainless steel^41^. In contrast to the distribution of Fe and Ni, the intensity of the metallic chromium (Cr_2_^−^) signal keeps high value within the oxide layer region, suggesting the enrichment of Cr on the stainless-steel surface by the laser filament processing.

We thus attribute the ultrahigh anticorrosion stainless steels to the synergistic effects of the resultant µm-thickness passivation layer with more abundant chromium contents and the unique hierarchically heterogeneous Cassie state having the sub-microstructures embedded in the microstructures with the flat-top shapes, which creates a thin air layer on the stainless-steel surface. Furthermore, we perform laser filament processing for the other stainless-steel surfaces of AISI 316, 420, 201, 430, and 2205. As shown in Fig. S15, the corrosion performances of the laser-filament processed stainless-steel surfaces under the saline, acidic, and alkaline conditions are significantly improved in all the cases, that is, their *i*_corr_ values are smaller than those of the corresponding unprocessed samples by 3-6 orders of magnitude. We can conclude that the SLF processing is a promising and universal approach to the fabrication of super anticorrosion stainless steels. Furthermore, we have also examined its availability and applicability to other alloys (titanium alloy Tc4) and metals (pure aluminum), and the results (Fig. S16) manifest that the SLF technique possesses a certain degree of universality in its applicability to different metal materials, but its effectiveness is still influenced by the chemical properties of the metals themselves. With recent advances in high-repetition-rate high-energy femtosecond lasers, it is anticipated that the SLF fabrication can be operated with much higher efficiency on a variety of irregularly shaped surfaces (see e.g., Fig. S17) by designing the filamentation to occur over a far distance in the atmosphere for practical applications in industry.

## Materials and methods

### Laser treatment

The schematic diagram of the experimental setup for the steel surface processing is shown in Fig. S1. A commercial Ti: Sapphire laser system (Spectra−Physics, Spitfire ACE) produced a linearly polarized laser pulse train with a central wavelength of 800 nm, a pulse width of ~35 fs, and a repetition rate of 1 kHz. The energy of the laser pulse was controlled to be 1.6 mJ by using a half wave plate and a polarizer. The laser pulse was focused by a fused silica lens (*f* = 100 cm) to generate a single filament with a length of about 4 cm and a diameter of 180∼190 μm. The laser intensity inside the generated filament was kept at 50-100 TW·cm^−2^. The strong-field laser filament then interacted with a steel target surface at a normal incident angle. In the experiment, the steel (AISI 304, 316, 420, 201, 430, and 2205), titanium alloy (Tc4,) and pure Al samples had a size of 10 mm × 10 mm and a thickness of 1 mm, and the spoons were made of 304 stainless steels. The samples were first polished by a series of abrasive papers with the grit numbers of 1000, 2000, 5000, and 7000, respectively, and then cleaned by ultrasonic waves successively in acetone, ethanol and deionized water, each for 15 minutes. After cleaning, the samples were dried for 15 min in a vacuum drying oven at 80 °C. The sample was mounted on a two-dimensional electric moving stage, and raster−scanned with a spacing of 100 µm between the two adjacent scanning lines. The scanning speed was set at 0.5 mm∙s^−1^, which corresponds to 200 laser pulses hitting on the same position for all the samples. This setting of the laser scanning speed is determined by examining the anticorrosion performance of the steels processed with different scanning speeds (see Fig. S18).

### Temporal evolutions of laser-induced shock wave and plasma plume

We carried out the measurement of the laser filament-induced shock wave using a shadowgraph method with a 532 nm Nd:YAG laser having a pulse duration of 100 ns and a beam diameter of 4 cm (Fig. S1). The laser beam propagated in a direction parallel to the sample surface and passed perpendicularly through the plasma generated by the interaction of the laser filament with the sample. The laser beam was then detected by an ICCD camera (Andor iStar) equipped on a spectrometer, whose entrance slit width was set at 2.5 mm and grating at the zero order, so that the shock wave shadowgraph images were directly taken by the ICCD. A fused silica lens (*f* = 6 cm) was inserted in the collection system to make the shock wave shadowgraph images on the ICCD have a higher resolution. The gate width of the ICCD was Δ*t* = 50 ns, and the gate delay of the ICCD was changed from *t* = 1 μs to *t* = 5 μs. Note that the arrival time of the laser pulse on the target was *t* = 0 μs. The shock wave patterns shown in Fig. 1 in the main text at the delay times of 1, 2, 3, 4, and 5 μs were accumulated over 340 laser shots. In the measurement of the temporal evolution of filament-induced plasma plume, we blocked the Nd: YAG laser, and directly detected the optical emissions of the plasma by the ICCD without changing the settings of the entrance slit and the grating of the spectrometer. The gate delay was changed from *t* = 0, 50, 100, 200, 400, 600 to 800 μs with variable gate widths of 1, 50, 50, 50, 50, 50, and 50 μs. The data were respectively accumulated over 40, 40, 40, 100, 2000, 20,000, and 20,000 laser shots in order to obtain high signal-to-noise ratios.

### Optical emission spectroscopy of filament-induced plasma

We used the same abovementioned spectrometer to measure the optical emission spectroscopy of filament-induced plasma from the target. In this measurement, the entrance slit of the spectrometer was set at 100 μm, and the grating was set at first order. The gate width and delay of the ICCD were adjusted to 50 ns and 500 ns, respectively.

### Surface treatments after the laser processing

The processed samples were cleaned by the ultrasonic method in deionized water for 15 min, and then dried for 15 min in a vacuum drying oven at 80 °C. The micro/nanostructured steel samples were further treated to reduce the surface energy by a low-temperature heating treatment, in which the samples were put in a heat oven at 150 °C for 2 h.

### Electrochemical measurements

The electrochemical measurements were conducted using a plate corrosion tank (Corrtest, CS936) and an electrochemical workstation (Gamry Reference 600, America) in a 3.5 wt.% NaCl aqueous solution, in a pH=2 HCl solution or in a pH=12 NaOH solution at 20 °C. The electrochemical measurements were performed in a typical three-electrode configuration, where the sample surface serves as the working electrode, a silver/silver chloride (Ag/AgCl) as the reference electrode, and a Pt mesh as the counter electrode. The electrochemical impedance spectroscopy (EIS) was performed in the frequency range from 10^5^ to 10^−2^ Hz at a stable state achieved after 2 h by monitoring the open circuit potential (OCP), and the alternating current amplitude was set at 10 mV. Then the EIS results were fitted with the ZSimPWin software. In order to obtain more reliable results, the measurements of the anodic and cathodic potentiodynamic polarization (PDP) curves were carried out, respectively, with the sample serving either as the anodic electrode or as the cathodic electrode. For the temperature-regulated electrochemical experiment, the PDP curves were obtained from the measurements conducted in the 3.5 wt.% NaCl aqueous solution at 20 °C, 30 °C, 40 °C, 50 °C and 60 °C, respectively. The anodic and cathodic PDP curves were acquired with a scanning speed of 1 mV·s^−1^. The corrosion current density (*i*_corr_) and corrosion potential (*E*_corr_) were calculated based on the Tafel extrapolation method from PDP curves by a CHI604E Electrochemical Analyzer software. For the multiple cycles PDP measurement, the experiment was performed in situ on the same sample with 2 h between the two adjacent cycle tests in order to obtain a stable open circuit potential. Cyclic voltammetry was carried out from 0 V to 0.1 V at the scan rates of 20 mV·s^−1^, 40 mV·s^−1^, 60 mV·s^−1^, 80 mV·s^−1^, 100 mV·s^−1^ and 200 mV·s^−1^, respectively. It should be pointed out that all the samples were kept under the same test conditions.

### Corrosion rate (CR) calculation

The corrosion rate was calculated by the following equation,

$${CR}=\frac{K\times {i}_{{\rm{corr}}}\times {EW}}{\rho }$$

where *EW* = 28.25 g and *ρ* = 7.85 g·cm^−3^ represent the equivalent weight and density of the 304 stainless steel samples, respectively; *K* = 3.273 × 10^−3^ mm·g·μA^−1^·cm^−1^·yr^−1^ is a conversion factor^17^.

### Equivalent electrical circuits

According to EIS measurements (Nyquist and Bode plots), the equivalent electrical circuits fitted for the pristine and the filament−processed samples are illustrated in Fig. S4a and S4b. In the equivalent electrical circuits, *R*_*s*_ denotes the solution resistance from the reference electrode to the working electrode, *R*_*f*_ is the resistance of the superhydrophobic film, *R*_*ct*_ the charge transfers resistance of the electrode, and *W*_*o*_ the Warburg impedance of solid phase diffusion. Constant phase element (CPE) was used to simulate the capacitance. The impedance of the CPE was calculated with the following equation^17,31^,

$${Z}_{{\rm{CPE}}}={Y}^{-1}{(j2{\rm{\pi }}f)}^{-n}$$

where *j* is the imaginary unit, *f* is the frequency, *Y* and *n* are the value and exponential coefficient associated with CPE, respectively. Under the experimental conditions, *CPE*_*dl*_ is the constant phase element associated with the electric double−layer capacitor, the value of which refers to the amount of corrosive ions through the surface film to touch substrates, and *CPE*_*f*_ is the constant phase element associated with the superhydrophobic film capacitor.

### Electrochemical surface area (ECSA)

The *C*_*dl*_ was estimated by plotting $\bar{j}=({j}_{a}+{j}_{c})/2$ at 0.05 V (where ${j}_{c}$ and ${j}_{a}$ are the cathodic and anodic current densities, respectively) as a function of the scan rate. To obtain the electrochemical surface area (ECSA), the roughness factor (*R*_*f*_) of the as−prepared electrodes can be acquired according to the following equation,

$${\rm{ECSA}}={R}_{f}\times S$$

where S stands for the geometric area (*S* = 0.785 cm^2^). According to the double-layer capacitance (*C*_*dl*_) of a smooth metal surface per square centimeter (20 μF·cm^−2^), *R*_*f*_ was calculated using the following equation^36^,

$${R}_{f}=\frac{{C}_{{dl}}}{20\,{\rm{\mu }}{\rm{F}}\,{{\cdot }}{{\rm{cm}}}^{-2}}$$

### Activation Energy (*E*_a_)

The activation Energy *E*_a_ of LH304 in the 3.5 wt.% NaCl aqueous solution was obtained from the linear variation of the corrosion current density with temperature by the following equation^42^,

$${\mathrm{lg}}{i}_{{\rm{corr}}}={\mathrm{lg}}{\rm{A}}-\frac{{E}_{a}}{2.303{RT}}$$

$$\frac{{\rm{d}}\,{\mathrm{lg}}{i}_{{\rm{corr}}}}{{\rm{d}}(\frac{1}{T})}=\frac{{E}_{a}}{2.303R}$$

where R stands for the universal gas constant (*R* = 8.314 J·mol^−1^·K ^−1^), *A* is the frequency factor and *T* is the absolute temperature.

### Materials characterizations

The surface morphologies of the steel samples were analyzed by a helium ion microscope (Zeiss, Orion NanoFab), a field emission scanning electron microscope (SEM) (JEOL, JSM-7500F), and a scanning laser confocal microscope (LSCM) (Olympus, OLS4100). A transmission electron microscope (FEI Talos, F200X G2) operating at 200 kV energy was used to measure the transmission electron microscopy (TEM), high-resolution TEM (HRTEM) images, and energy dispersive spectroscopy (EDS). The thin slices for TEM test were cut by a focused ion beam (FIB) equipment (FEI, Scios2). The X-ray photoelectron spectroscopy (XPS) patterns were measured using a monochromatic Al Kα X-ray source (hν = 1486.6 eV) and were analyzed using the Avantage v5.9931 software. The XPS and UPS depth profiles were measured by a self- assembled system including an electron analyzer (Omicron, EA125) equipped with an Al Kα X-ray source (Omicron, DAR400) (*hν* = 1486.7 eV) for XPS and a He discharge lamp (He 1α at *hν* = 21.2 eV) (Omicron, VUV HIS 13) for UPS, and the sputtering was conducted using an Argon ion sputtering gun with an operation energy of 1.0 KeV at the Argon pressure of 5.0 × 10^−5^ mbar. Time-of-flight secondary ion mass spectrometry (ToF-SIMS) analysis was performed using a ToF-SIMS 5 spectrometer (TOF.SIMS 5, ION-TOF GmbH) operating at a pressure of 2.0 × 10^−8^ mbar. Topmost surface analysis in static SIMS conditions was performed using a pulsed 30 keV Bi^+^ primary ion source delivering 3 pA current over a 50 × 50 μm^2^ area, and then was interlaced with sputtering using a 2 keV Cs^+^ ion beam giving a 70 nA target current over a 200 × 200 μm^2^ area. The compositions of the samples surface were also analyzed by an X-ray diffraction spectrometer (XRD) (Rigaku, Uiltia IV).

### Contact angle and rolling angle test

The water contact angles (CA) and rolling angles (RA) of the steel surfaces were measured in a static manner at 20 °C by using a contact angle tester (INNUO, CA100D) with a 10 μL distilled water droplet.

### Friction coefficient test

A high temperature friction and wear testing machine ((Lanzhou Zhongke Kaihua Technology Development Co., Ltd., HT-1000) was used to perform a 30-min friction test, respectively on the pristine sample and LH304 under the conditions of a rotational speed of 200 rpm and a normal load of 200 N.

### Simulated rainfall test

A showerhead with a nozzle diameter of 1 mm was used to simulate rainfall. Water droplets impacted the LH304 surface at a speed of 1 m ∙ s^−1^, with an approximate volume of ~0.3 mL per droplet.

### Sandpaper abrasion test

The LH304 was placed face-down on 4000-mesh sandpaper and subjected to horizontal abrasion under a 100 g load for 5 cycles. Each cycle involved a 20 cm linear displacement.

### External compression test

The LH304 was placed face-up on a flat surface, and a load of 5 kg was applied for 2 h.

### Wave-simulated vibration test

The LH304 was placed in a glass tank filled with 3.5 wt.% NaCl aqueous solution, where continuous wave motion was generated by a water pump. The sample was exposed to this environment for 10 hours to simulate the erosive effect of flowing water on the surface under real-world conditions.

### Ultrasonic cleaning test

The LH304 was placed in an ultrasonic cleaner and treated for 10 minutes at 4000 kHz.

## Supplementary information

Supplementary information

## Data Availability

The data that support the findings of this study are available from the corresponding author upon reasonable request.
