# Supplementary material for: Significant reduction of corrosion of stainless steel by strong-field laser surface passivation
Source: Light Sci Appl. 2025 Oct 5;14:352. doi: 10.1038/s41377-025-01952-5 (PMC12496454; doi:10.1038/s41377-025-01952-5)
Supplement: Supplementary file 1 — Supplementary information [file 41377_2025_1952_MOESM1_ESM.docx]

**Supplementary Information**

**Significant reduction of corrosion of stainless steel by strong-field laser surface passivation**

Liansheng Zheng^1+^, Hongwei Zang^1,2+^, Yuan Liu^3,4^, Yukun Xiao^3,4^, Yingbo Cong^5^, Zhen Cheng^6^, Ganwen Chen^3,4^, Zhenxiang Xing^7^, Jisheng Pan^7^, Qing Jiang^8^, Wei Chen^3,4^, Kaoru Yamanouchi^9^, Huailiang Xu^1,10^*, and Ruxin Li^2,11^*

* These authors are corresponding authors:

Huailiang Xu, Email: huailiang@jlu.edu.cn; Telephone: +86 431 85168272

Ruxin Li, Email: ruxinli@mail.siom.ac.cn; Telephone: +86 21 20685319

^+^These authors contributed equally to this work.

Table of contents

Supplementary Figure 1-18

Supplementary Table 1-6


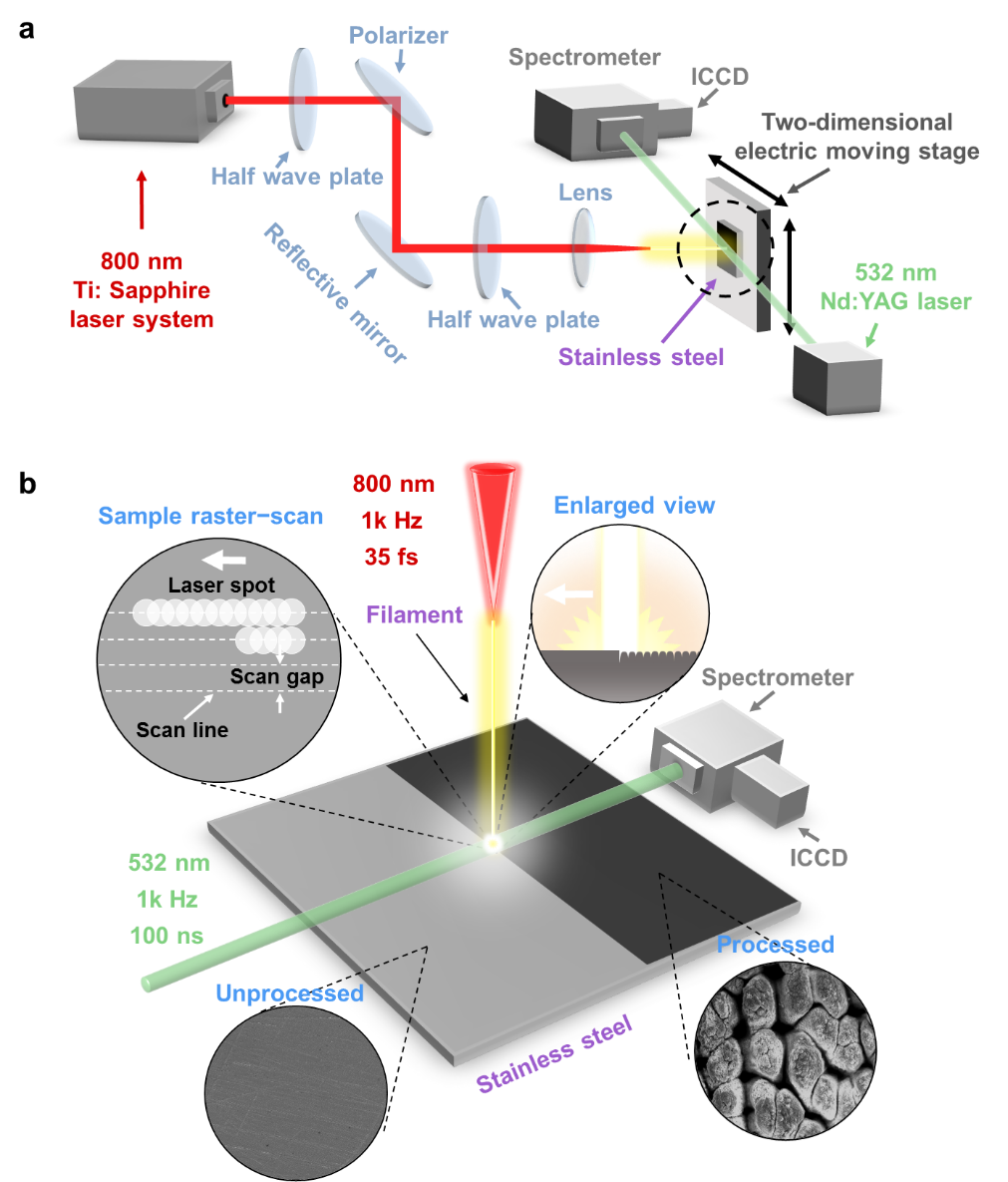


**Supplementary Figure 1 ⏐ Strong-field laser processing of stainless steel surfaces with a femtosecond filament.**

**a** Schematic of the optical setup for strong-field laser processing of stainless steel surfaces. **b** Schematic for the raster-scan processing, and the measurements of temporal evolutions of laser-induced shock wave and plasma plume, as well as optical emission spectroscopy of filament-induced plasma.

**
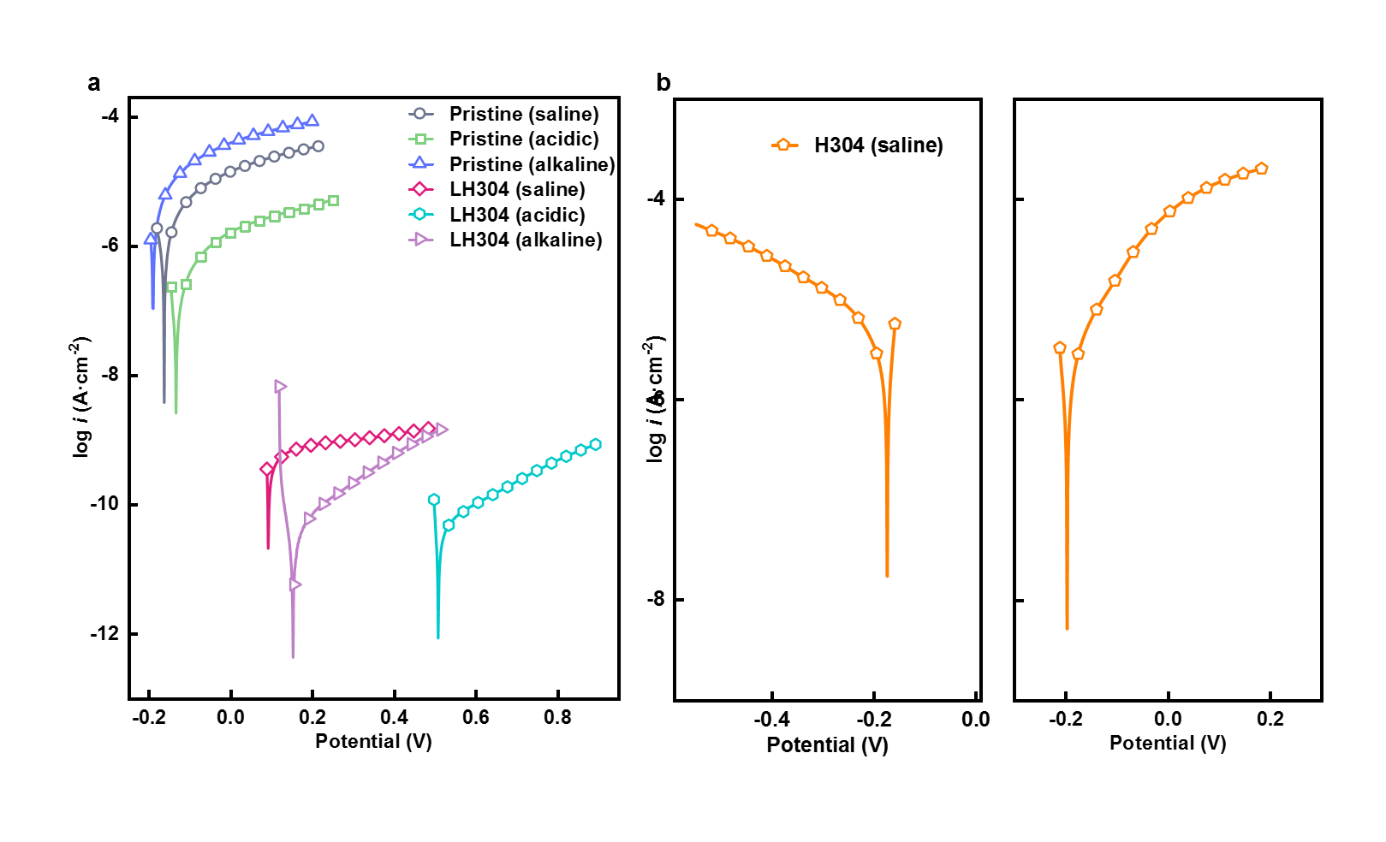
**

**Supplementary Figure 2 ⏐ Cathodic and anodic PDP curves of steel surfaces under different conditions.**

**a** The anodic PDP results of the pristine samples and LH304 samples performed in saline (3.5 wt. % NaCl), acidic (HCl, pH=2) and alkaline (NaOH, pH=12) solutions, respectively. **b** The cathodic and anodic PDP measurement of the heat-only treated 304 stainless steel samples in the 3.5 wt. % NaCl aqueous solution.


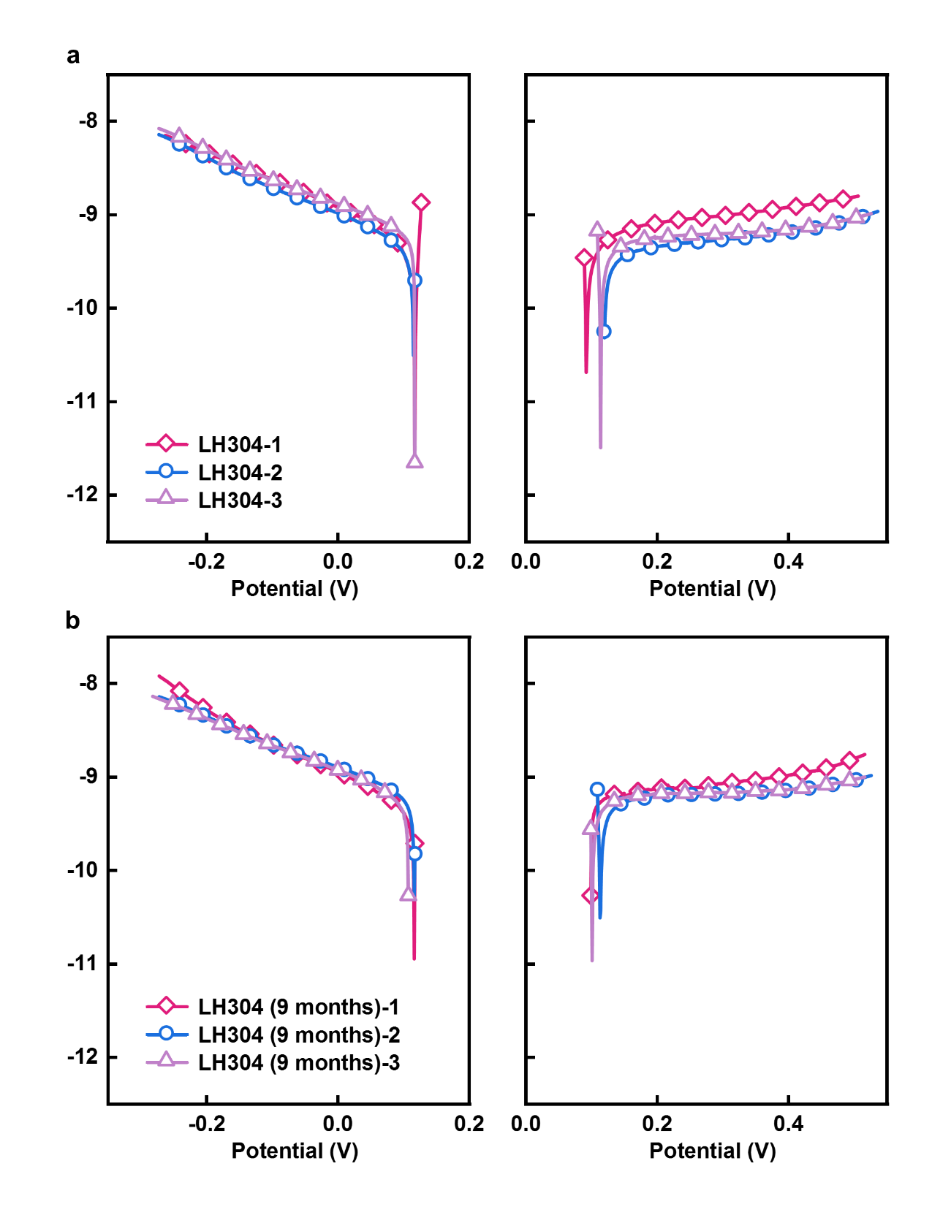


**Supplementary Figure 3 ⏐ The PDP curves of the LH304 samples and those immersed in the 3.5 wt. % NaCl aqueous solution for 9 months.**

**a** The cathodic and anodic PDP curves of the LH304 samples in the 3.5 wt. % NaCl aqueous solution. **b** The cathodic and anodic PDP curves of the LH304 samples immersed in the 3.5 wt. % NaCl aqueous solution for nine months. The consistence on the measured corrosion current densities of different fabricated samples indicates that the SLF processing technique possesses excellent reproducibility.

**
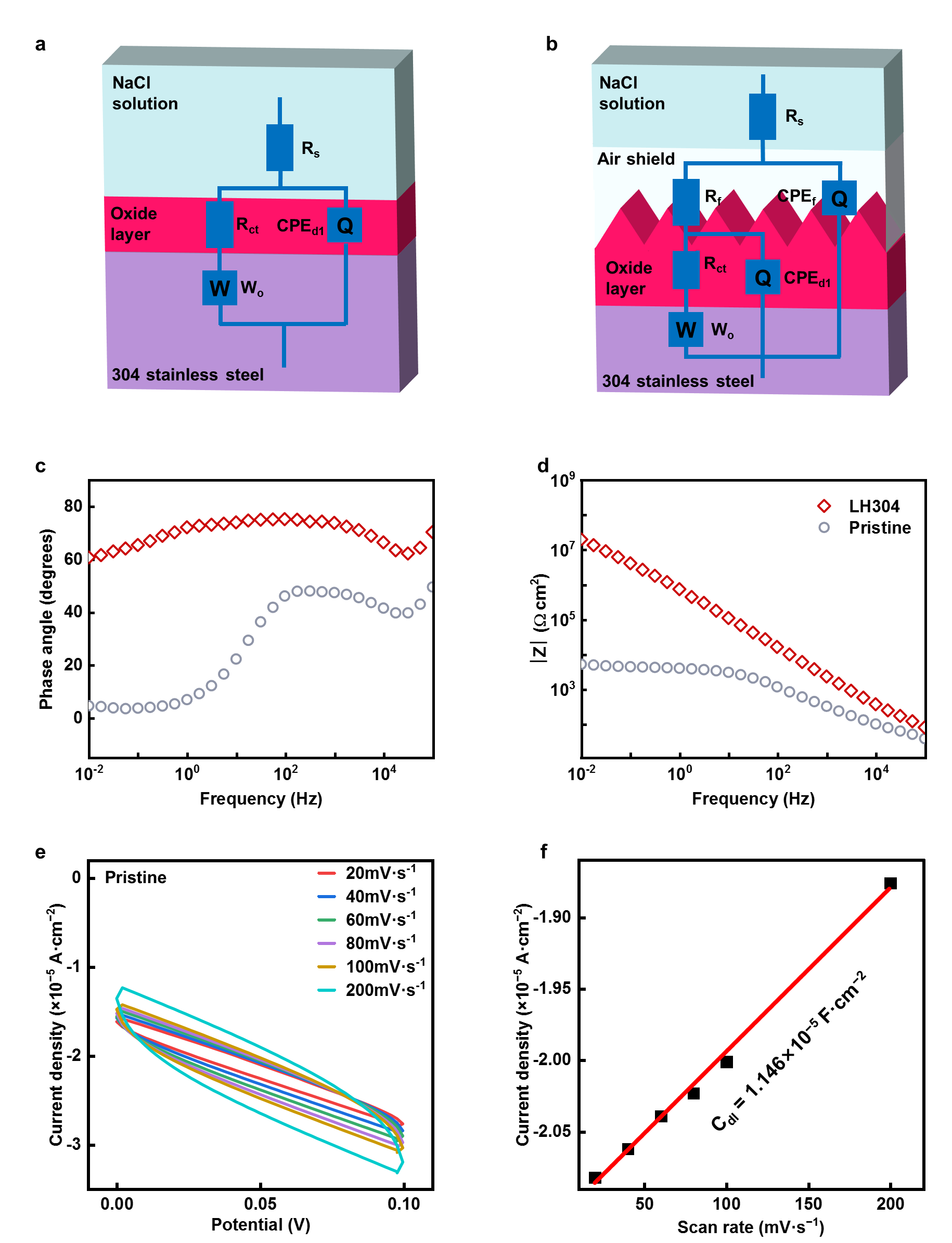
**

**Supplementary Figure 4 ⏐ Equivalent electrical circuits, bode plots and cyclic voltammograms.**

**a**, Equivalent electrical circuits for fitting EIS and bode plots of the pristine sample and **b**, Equivalent electrical circuits for fitting EIS and bode plots of the processed LH304 samples. **c**, The phase angle in the Bode plots measured as a function of the frequency for the pristine (circles) and LH304 (diamonds) samples. **d**, The impedance modulus in the Bode plots measured as a function of the frequency for the pristine (circles) and LH304 (diamonds) samples. **e**, The measured cyclic voltammogram of the pristine sample. **f**, The measured (rectangle dots) and fitted (solid line) double layer charging current densities as a function of the scan rate of the pristine sample.


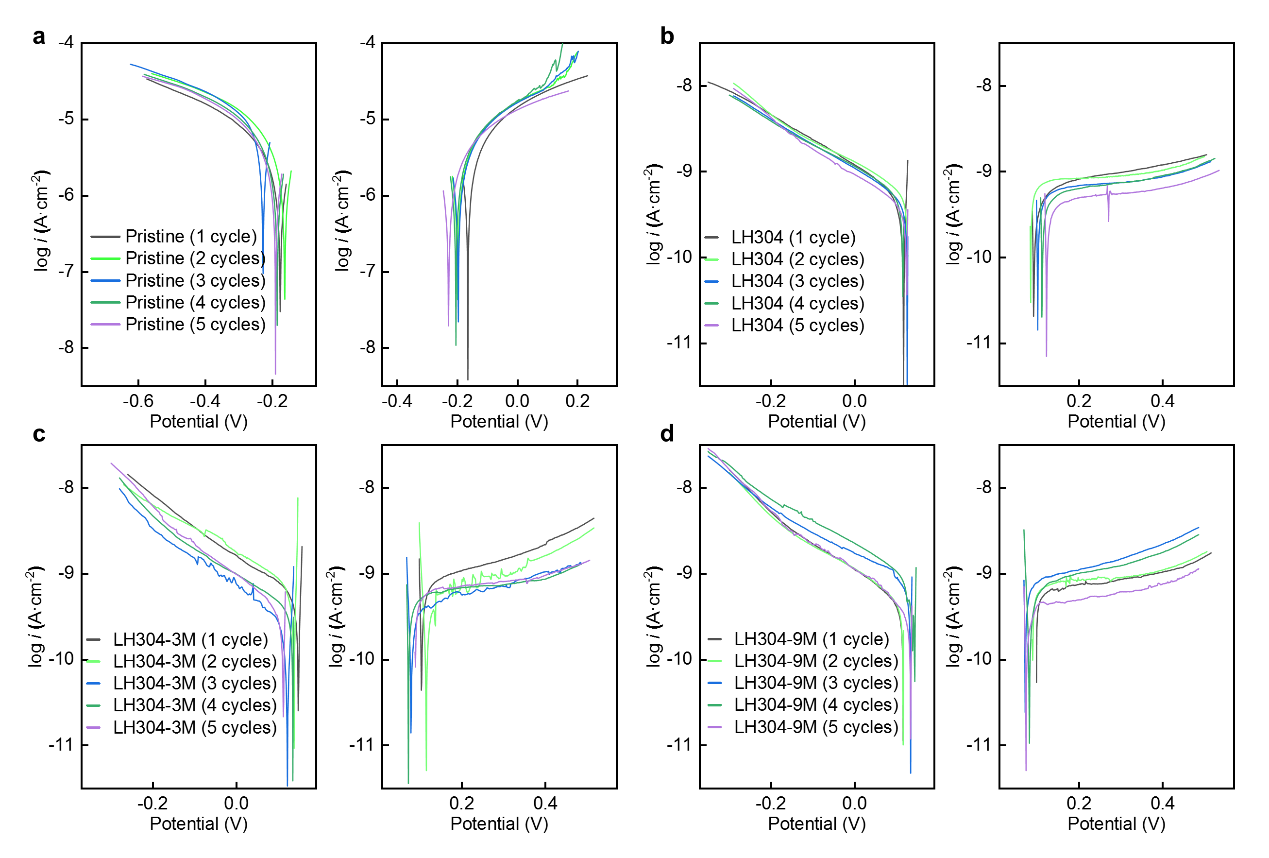


**Supplementary Figure 5 ⏐ Cathodic and anodic PDP measurements for characterizing the anticorrosion durability.**

The cathodic and anodi c PDP curves measured repeatedly from 1 to 5 cycles for the (**a**), pristine sample, (**b**), LH304 sample, (**c**), LH304 sample immersed in the 3.5 wt. % NaCl aqueous solution for three months, and (**d**), LH304 samples immersed in the 3.5 wt. % NaCl aqueous solution for nine months.


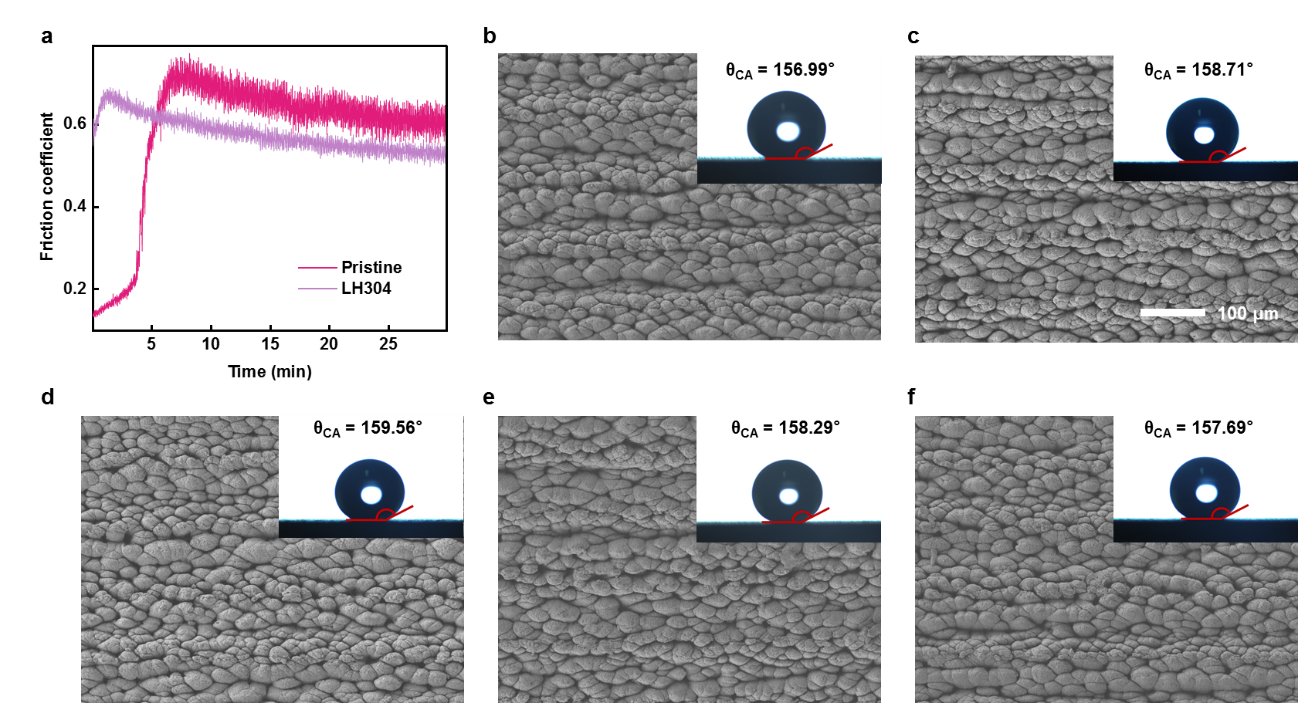


**Supplementary Figure 6 ⏐ Mechanical stability test results of the LH304 samples**

**a** Friction coefficient as a function of time. SEM images and static contact angles of the LH304 surface measured for (**b)** simulated rainfall, (**c**) sandpaper abrasion, (**d**) external compression, (**e**) wave-simulated vibration, and (**f**) ultrasonic cleaning tests, respectively.


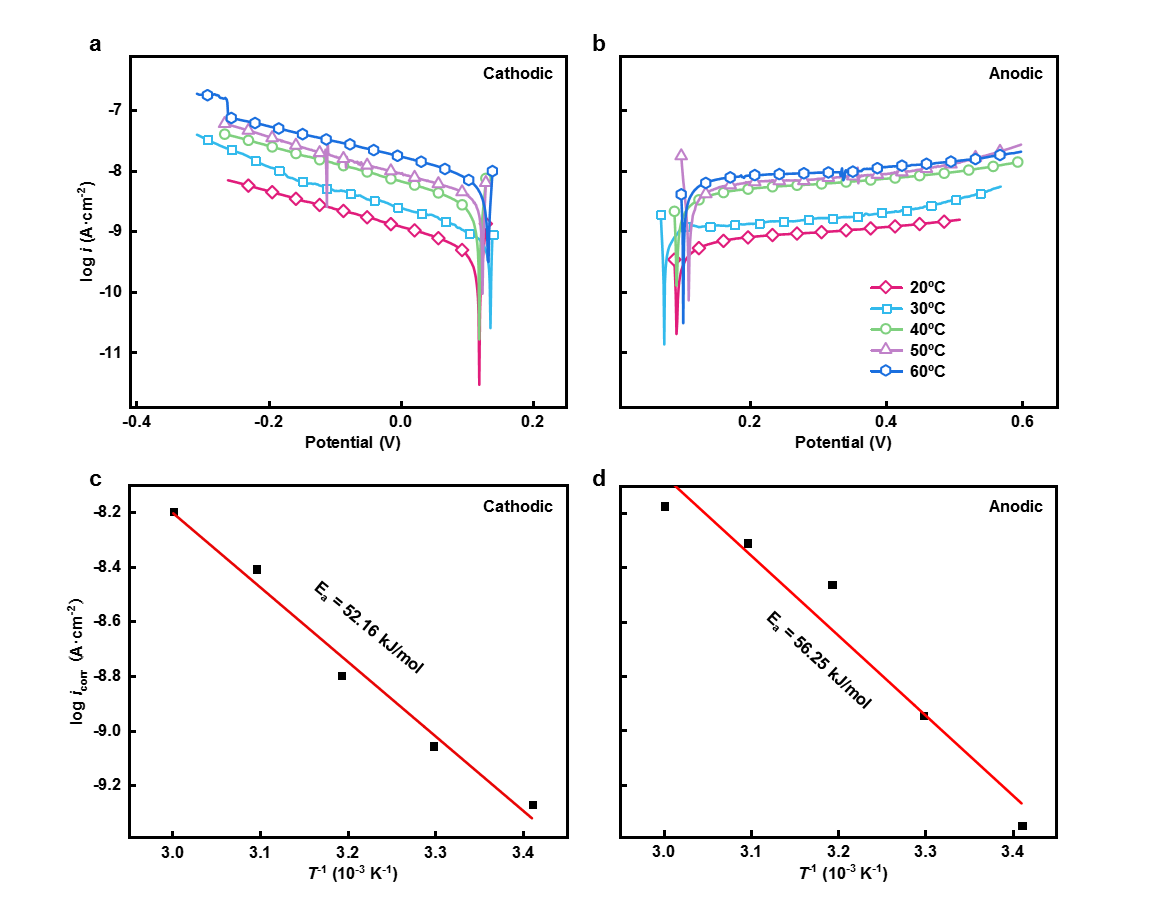


**Supplementary Figure 7 ⏐ The Arrhenius plot of LH304.**

Cathodic (**a**) and anodic (**b**) PDP curves of the LH304 samples measured in the 3.5 wt. % NaCl aqueous solution at 20 °C, 30 °C, 40 °C, 50 °C, and 60 °C. Arrhenius plot of the corrosion current density (log i_corr_) as a function of the reciprocal of temperature (T^-1^) for the LH304 surface of Cathodic (**c**) and anodic (**d**) PDP curves.

**
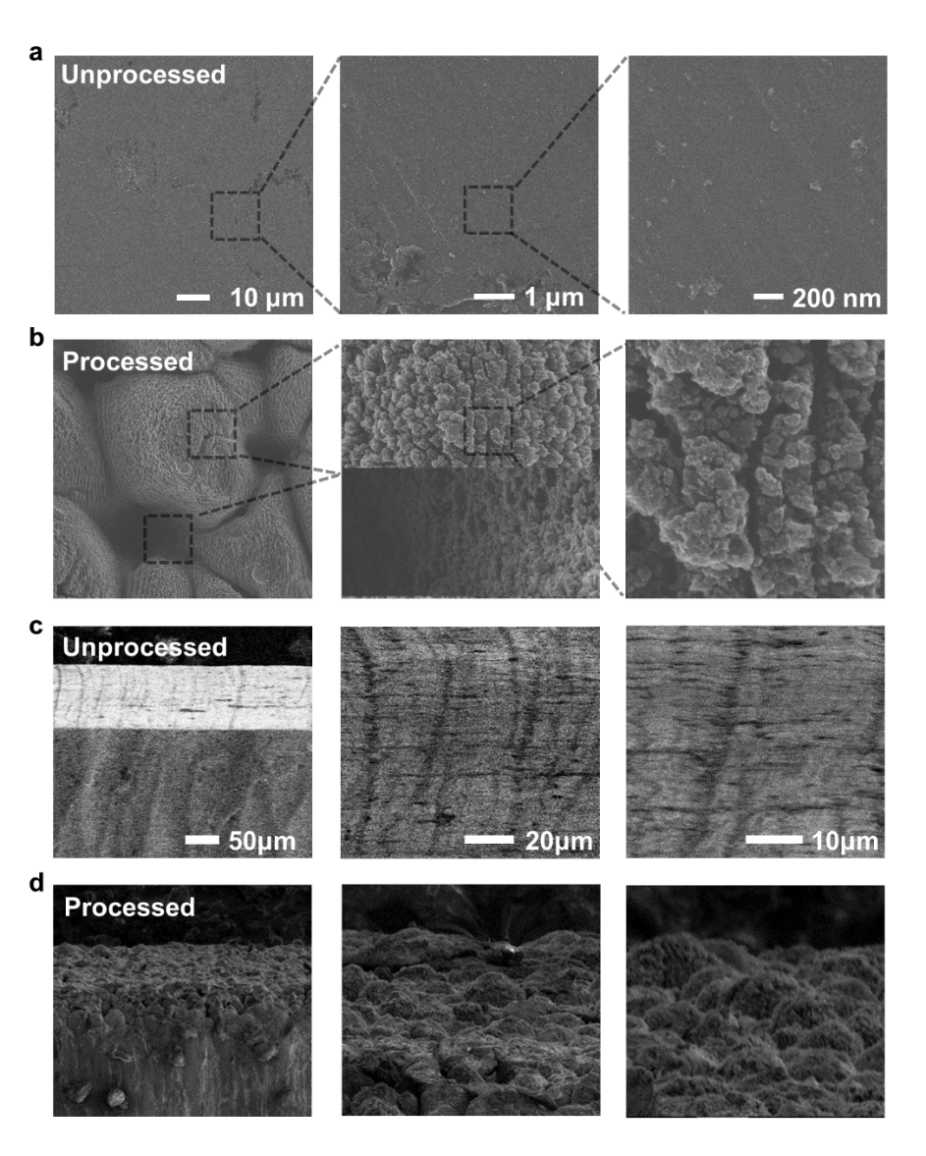
**

**Supplementary Figure 8 ⏐ Surface morphology images of the unprocessed and processed samples.**

Surface morphology images of the (**a**) unprocessed and (**b**) processed samples measured by a helium ion microscope (Orion NanoFab) at different magnifications. Side-view SEM images of the unprocessed (**c**) and processed (**d**) sample surfaces measured by a scanning electron microscope (JEOL JSM-7500F) at different magnifications.


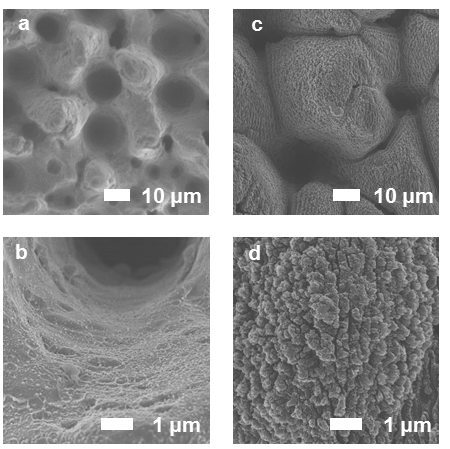


**Supplementary Figure 9 ⏐ The HIM images of surface resulting from single-spot ablation and scanning processing.**

Surface images obtained by helium-ion microscope (HIM) showing surface morphologies resulting from (**a**, **b**) single-spot ablation and (**c**, **d**) raster-scan processing, respectively. The hierarchically heterogeneous micro-nanostructures are mainly due to the unique energy distribution of the filament, where a high-intensity filament core surrounded by a weak-intensity energy reservoir. That is, the filament core induces the microstructures and the energy reservoir produces the nanostructures.


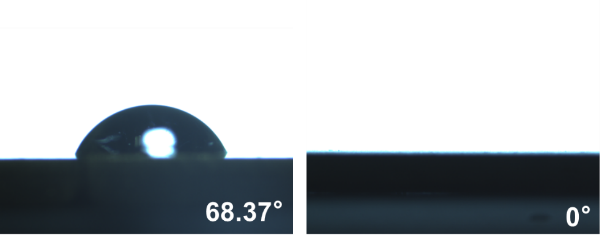


**Supplementary Figure 10 ⏐ Water Contact angles (CAs).**

Water CAs measured by a contact angle tester (INNUO, CA100D) for the H304 (left) and L304 (right) samples.


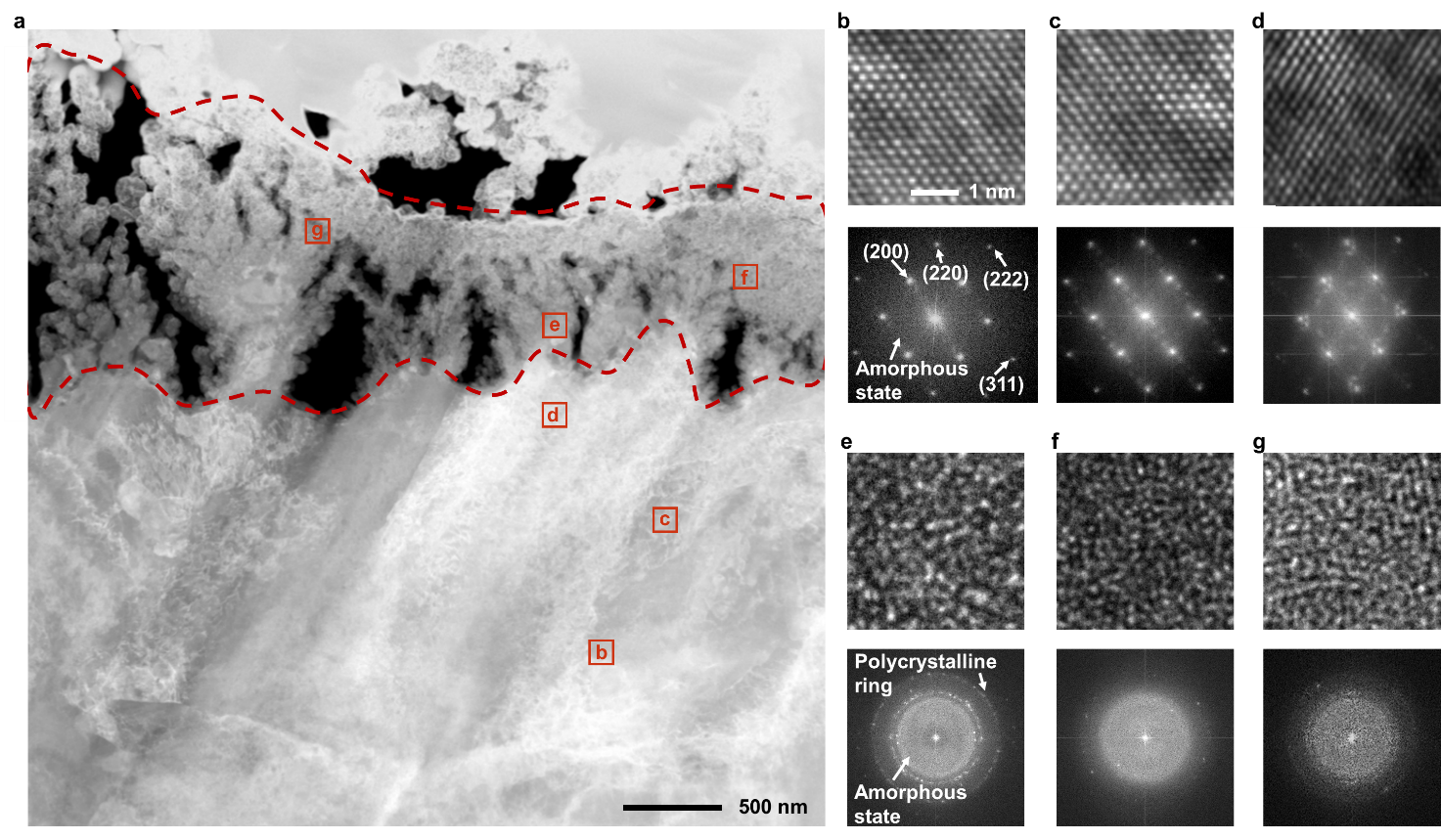


**Supplementary Figure 11 ⏐ HRTEM of different place.**

**a** Dark-field TEM of the LH304 surface. **b**-**g** HRTEM and fast Fourier transform pattern of different place.


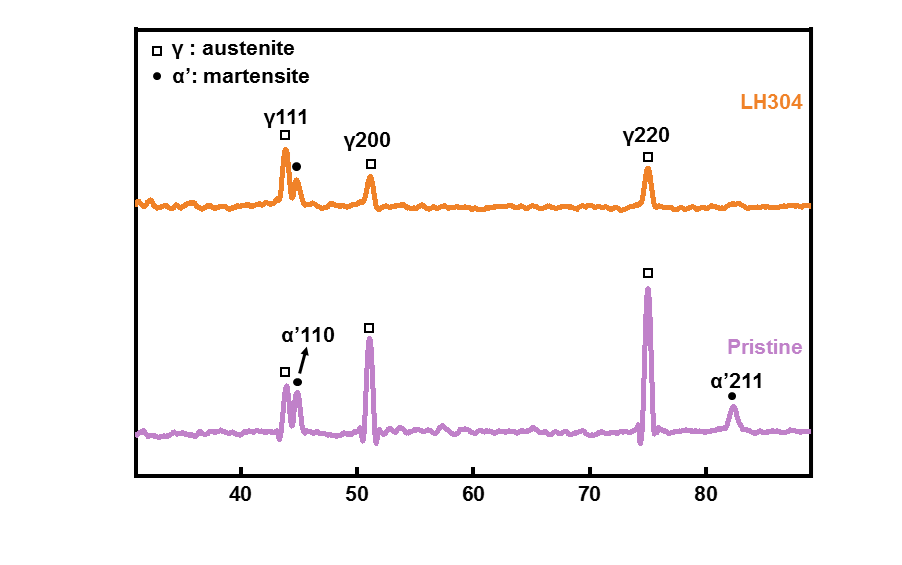


**Supplementary Figure 12 ⏐ XRD spectra.**

XRD spectra of the LH304 (yellow) and pristine samples (purple). γ (γ: 111 at θ=43.65°, 200 at 50.76° and 220 at θ=74.58°) and α’ (α’: 200 at θ=44.8°, 211 at θ=64° and 110 at θ=82.3°) correspond to the austenite and martensite structures, respectively.

**
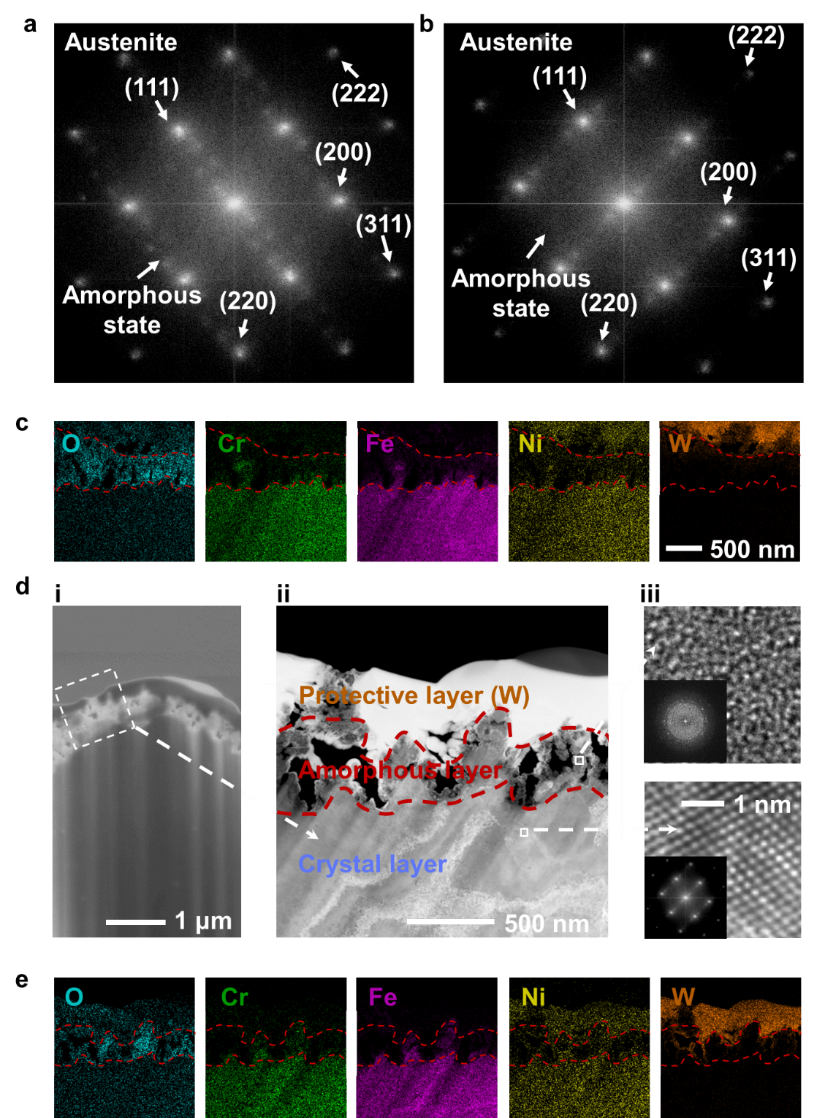
**

**Supplementary Figure 13 ⏐ TEM, EDS and HRTEM.**

Fast Fourier transformation of HRTEM for (**a**) LH304 and (**b**) L304. The measured diffraction spots match the (111), (222), (200), (311) and (220) crystalline planes of austenite and also the diffusion halo of amorphous state. **c**, High-resolution EDS results of LH304. **d** Images of (**d_i_**) the L304 sample prepared by FIB, (**d_ii_**) dark-field TEM, and (**d_iii_**) HRTEM (Inset: fast Fourier transform (FFT) pattern). (**e**) High-resolution EDS results of L304.

**
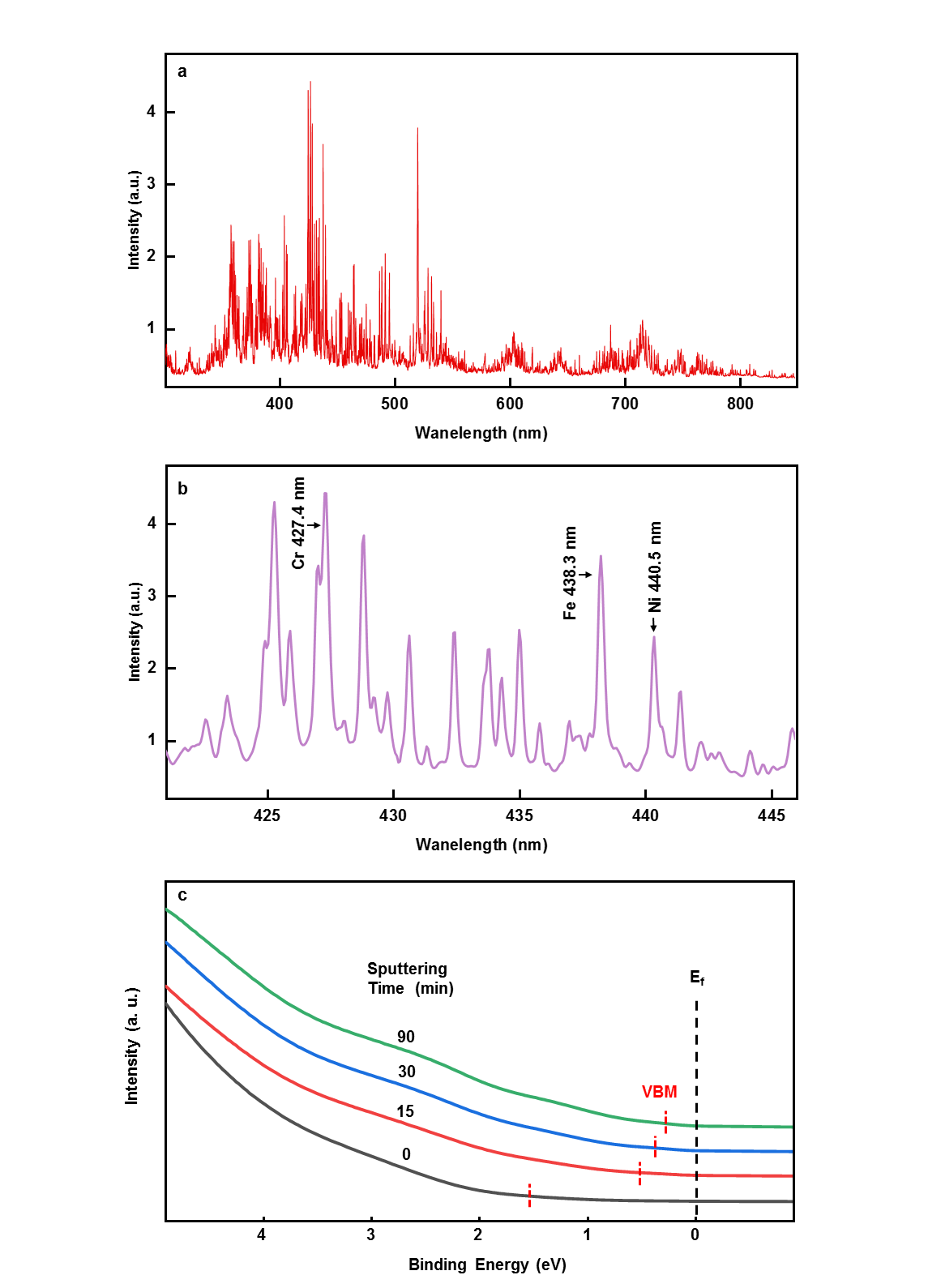
**

**Supplementary Figure 14 ⏐ The optical emission spectroscopy and Valence-band UPS spectra of L304.**

The optical emission spectroscopy of filament-induced plasma measured from the surface of the 304 sample in the range of (**a**) 300-850 nm and (**b**) 421–446 nm, respectively. **c**, Valence-band UPS spectra close to the Fermi edge of the L304 obtained after consecutive sputtering for 0, 15, 30, and 90 min.


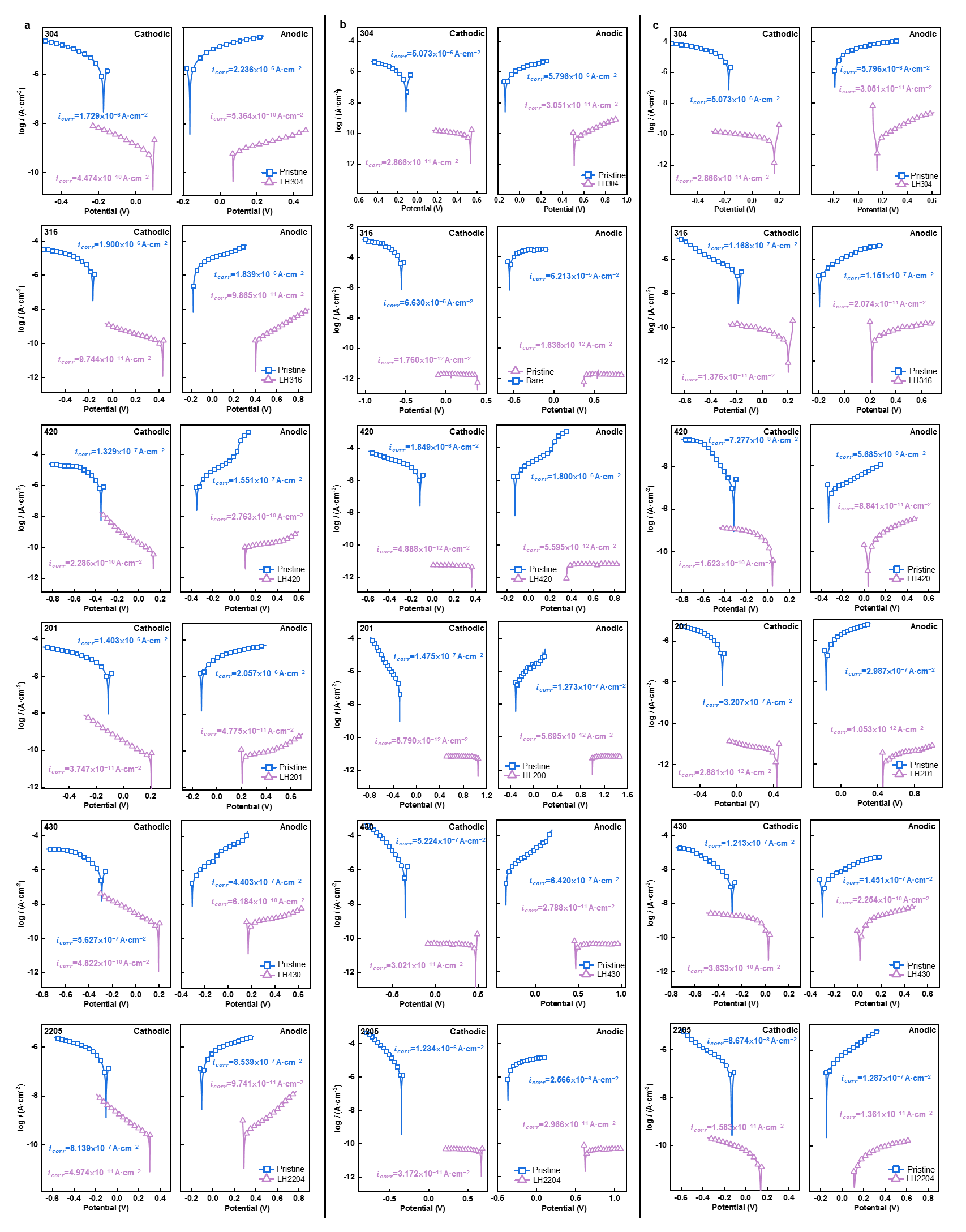


**Supplementary Figure 15 ⏐** **The anodic and cathodic PDP curves of different types of steels.**

The cathodic and anodic PDP curves of the pristine and processed 304, 316, 420, 201, 430 and 2205 stainless steel samples measured in the (**a**) 3.5 wt. % NaCl, the (**b**) pH=2 HCl and the (**c**) pH=12 NaOH aqueous solution.


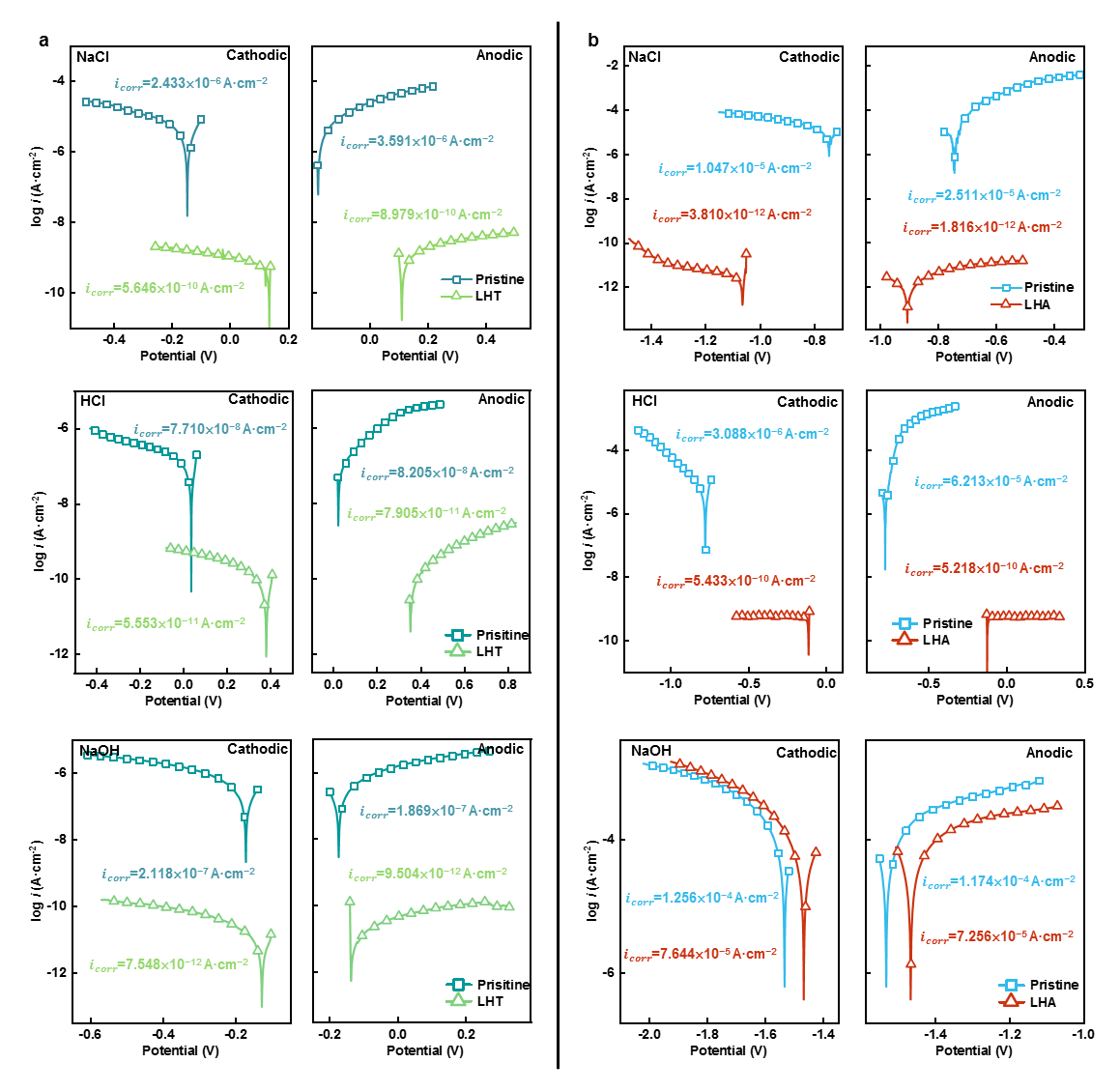


**Supplementary Figure 16 ⏐ The anodic and cathodic PDP curves of different metals.**

The cathodic and anodic PDP curves of the pristine and processed (**a**) Tc4 and (**b**) Al samples measured in the 3.5 wt. % NaCl, the pH=2 HCl and the pH=12 NaOH aqueous solution.


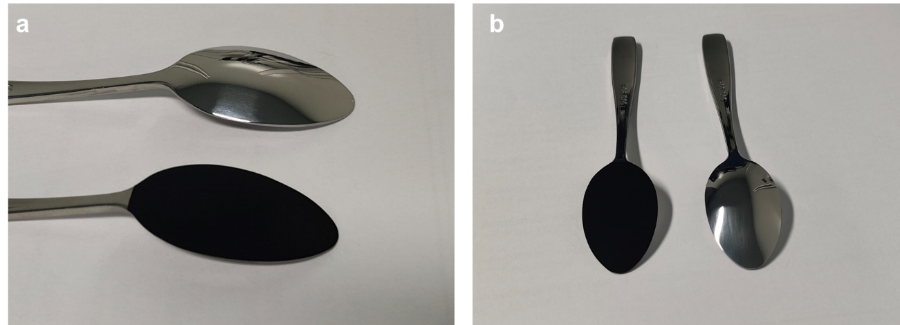


**Supplementary Figure 17 ⏐ The filament-processed spoons.**

Photographs of the side view (**a**) and front view (**b**) of 304 stainless-steel spoons before and after femtosecond laser filament processing.

**
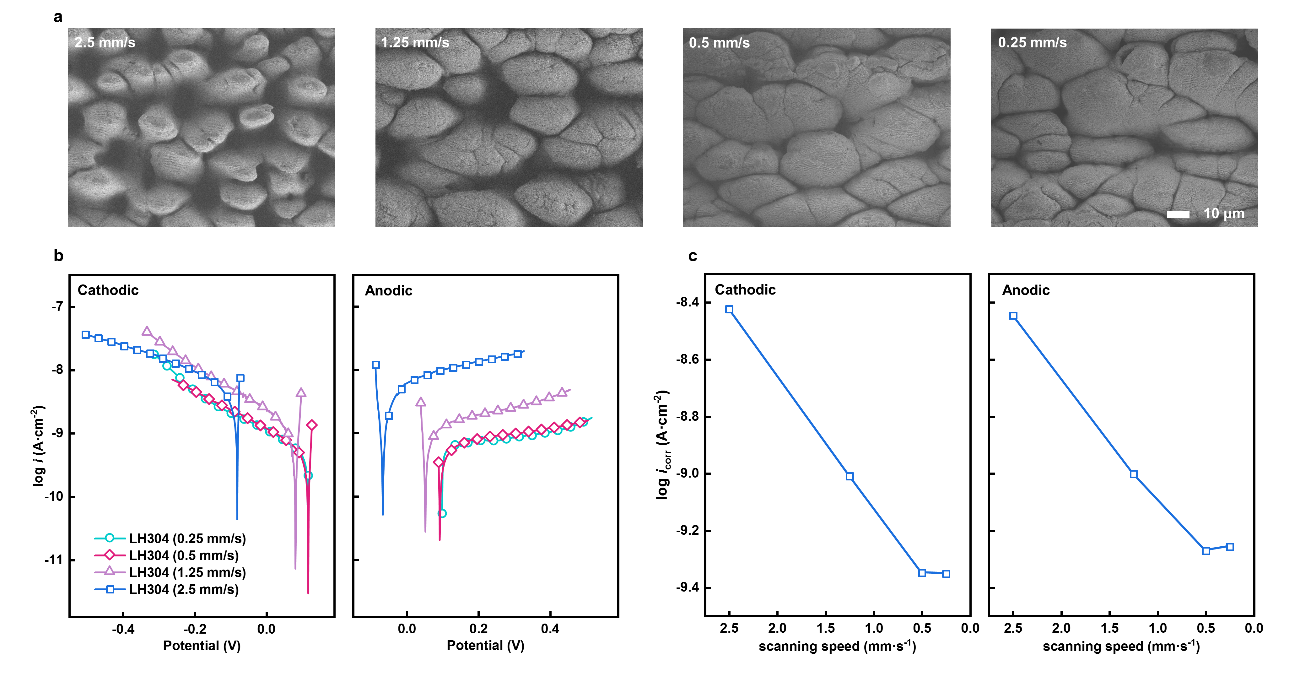
**

**Supplementary Figure 18 ⏐ The SEM imagines and PDP curves of LH304 surfaces processed by the laser filament with different scanning speeds.**

(**a**) The SEM images, and (**b**) the cathodic and anodic PDP curves of the 304 stainless steels surfaces processed by the laser filament with different laser shots of 40, 80, 200 and 400 hitting on the same position, which correspond to the scanning speeds of 2.5, 1.25, 0.5, and 0.25 mm s^-1^, respectively. **c** the dependence of *i*_corr_ on the laser pulse number. The corrosion current density *i*_corr_ of the fabricated stainless steels first decreases as the number of the laser shots increases (i.e., as the scanning speed decreases), and then keep almost unchanged after the number of the laser shots reaches 200 (the scanning speed: 0.5 mm s^-1^), which shows the significance of optimizing the scanning speed to achieve the optimal anticorrosion effect.

**Supplementary Table 1. The corrosion parameters of the pristine, LH304 and H304 samples obtained from the recorded PDP curves.**

| Surface condition | $E_{\mathrm{corr}}$(V) | | $i_{\mathrm{corr}}$(A·cm^−2^) | | *CR* (μm·year^−1^) | |
| --- | --- | --- | --- | --- | --- | --- |
|  | Anodic | Cathodic | Anodic | Cathodic | Anodic | Cathodic |
| Pristine (saline) | −0.162 | −0.173 | 2.236×10^−6^ | 1.729×10^−6^ | 26.31 | 20.35 |
| Pristine (acidic) | -0.133 | -0.112 | 2.953×10^−7^ | 1.741×10^−7^ | 3.475 | 2.049 |
| Pristine (alkaline) | -0.189 | -0.171 | 5.796×10^−6^ | 5.073×10^−6^ | 68.19 | 59.69 |
| LH304 (saline) | 0.093 | 0.120 | 5.364×10^−10^ | 4.474×10^−10^ | 6.312×10^−3^ | 5.265×10^−3^ |
| LH304 (acidic) | 0.539 | 0.510 | 3.049×10^−11^ | 2.876×10^−11^ | 3.588×10^−4^ | 3.384×10^−4^ |
| LH304 (alkaline) | 0.154 | 0.165 | 3.051×10^−11^ | 2.866×10^−11^ | 3.590×10^−4^ | 3.372×10^−4^ |
| H304 (saline) | -0.196 | -0.171 | 2.082×10^−6^ | 3.369×10^−6^ | 21.75 | 35.20 |

**Supplementary Table 2. The corrosion parameters reported in the literatures.**

| Laser | Metal | CA (°) | Solution | Processed sample *i*_corr_ (A·cm^-2^) | Pristine sample *i*_corr_ (A·cm^-2^) | *i*_corr_ (pristine) / *i*_corr_ (laser processed) | References |
| --- | --- | --- | --- | --- | --- | --- | --- |
| fs | 304 | 157 | NaCl  (3.5 wt. %) | 1.22×10^−5^ | 5.75×10^−6^ | 0.47 | (*20*) |
|  | 304 | 144 | NaCl  (3.5 wt. %) | 1.27×10^−7^ | 7.6×10^−5^ | 59.84 | (*22*) |
| ns | 304 | 158.9 | NaCl  (3.5 wt. %) | 1.002×10^−7^ | 2.148×10^−6^ | 21.43 | (*23*) |
|  | 304 | 158.7 | NaCl  (3.5 wt. %) | 2.68×10^−6^ | 3.75×10^−5^ | 13.99 | (*17*) |
|  | 304 | 158.7 | Acidic  (pH=2) | 3.65×10^−7^ | 6.54×10^−5^ | 179.18 | (*17*) |
|  | 304 | 158.7 | Alkaline  (pH=12) | 1.45×10^−7^ | 7.6×10^−5^ | 524.14 | (*17*) |
|  | 316 | 168 | NaCl  (3.5 wt. %) | 2.9×10^−7^ | 7.2×10^−7^ | 2.48 | (*24*) |
|  | 316 | 160 | NaCl  (3.5 wt. %) | 2.8×10^−8^ | 2×10^−6^ | 71.42 | (*25*) |
|  | 316 | 158 | NaCl  (3.5 wt. %) | 4.7×10^−7^ | 4.6×10^−6^ | 9.78 | (*26*) |
|  | 316 | 157 | NaCl  (3.5 wt. %) | 4.2×10^−7^ | 3.2×10^−7^ | 0.76 | (*27*) |
|  | 316 | 154 | NaCl  (3.5 wt. %) | 2.2×10^−8^ | 1.6×10^−6^ | 72.73 | (*28*) |

**Supplementary Table 3. The corrosion parameters obtained for the LH304 samples of PDP measurements in the 3.5 wt. % NaCl aqueous solution, as well as for the LH304 samples immersed in the 3.5 wt. % NaCl aqueous solution for nine months.**

| Surface condition | | $E_{corr}$(V) | | $i_{corr}$(A·cm^−2^) | |  |
| --- | --- | --- | --- | --- | --- | --- |
|  |  | Anodic | Cathodic | Anodic | Cathodic |  |
| LH304 | Sample 1 | 0.093 | 0.120 | 5.364×10^−10^ | 4.474×10^−10^ |  |
|  | Sample 2 | 0.121 | 0.118 | 3.639×10^−10^ | 4.305×10^−10^ |  |
|  | Sample 3 | 0.115 | 0.120 | 4.721×10^−10^ | 6.176×10^−10^ |  |
|  | Average | 0.110 | 0.119 | 4.575×10^−10^ | 4.985×10^−10^ |  |
| LH304  (9 months) | Sample 1 | 0.101 | 0.119 | 5.438×10^−10^ | 4.469×10^−10^ |  |
|  | Sample 2 | 0.114 | 0.119 | 5.172×10^−10^ | 5.976×10^−10^ |  |
|  | Sample 3 | 0.102 | 0.110 | 5.621×10^−10^ | 5.601×10^−10^ |  |
|  | Average | 0.106 | 0.116 | 5.410×10^−10^ | 5.349×10^−10^ |  |

**Supplementary Table 4. EIS parameters calculated from the fitted plots of the pristine and LH304 samples, respectively.**

| Surface condition | *R_s_*  (Ω cm^2^) | *CPE_dl_*~*Y_dl_*  (Ω^−1^ cm^−2^ s^n^) | *CPE_dl_*~*n_dl_* | *R_f_*  (Ω cm^2^) | *CPE_f_*~*Y_f_*  (Ω^−1^ cm^−2^ s^n^) | *CPE_f_*~*n_f_* | *R_ct_*  (Ω cm^2^) | *W_o_*  (Ω^−1^ cm^−2^ s^0.5^) |
| --- | --- | --- | --- | --- | --- | --- | --- | --- |
| Pristine | 18.1 | 1.553×10^−5^ | 0.5954 | − | − | − | 4578 | 8.768×10^−3^ |
| LH304 | 10 | 2.823×10^−8^ | 0.9457 | 299.8 | 1.988×10^−7^ | 0.8118 | 6.919×10^6^ | 7.801×10^−8^ |

**Supplementary Table 5. The corrosion parameters obtained for the pristine and LH304 samples with different cycles of PDP measurements in the 3.5 wt. % NaCl aqueous solution, as well as for the LH304 samples immersed in the 3.5 wt. % NaCl aqueous solution for three and nine months.**

| Surface condition | | $E_{corr}$(V) | | $i_{corr}$(A·cm^−2^) | | *CR* (μm·year^−1^) | |
| --- | --- | --- | --- | --- | --- | --- | --- |
|  |  | Anodic | Cathodic | Anodic | Cathodic | Anodic | Cathodic |
| Pristine | 1 cycle | −0.162 | −0.173 | 2.236×10^−6^ | 1.729×10^−6^ | 26.31 | 20.35 |
|  | 2 cycles | −0.196 | −0.159 | 2.137×10^−6^ | 2.283×10^−6^ | 25.14 | 26.86 |
|  | 3 cycles | −0.194 | −0.224 | 2.137×10^−6^ | 5.614×10^−6^ | 25.14 | 66.06 |
|  | 4 cycles | −0.227 | −0.187 | 1.476×10^−6^ | 2.225×10^−6^ | 17.37 | 26.18 |
|  | 5 cycles | −0.202 | −0.182 | 2.085×10^−6^ | 2.276×10^−6^ | 24.53 | 26.78 |
| LH304 | 1 cycle | 0.093 | 0.120 | 5.364×10^−10^ | 4.474×10^−10^ | 6.312×10^−3^ | 5.265×10^−3^ |
|  | 2 cycles | 0.086 | 0.129 | 7.727×10^−10^ | 5.416×10^−10^ | 9.093×10^−3^ | 6.373×10^−3^ |
|  | 3 cycles | 0.103 | 0.129 | 5.654×10^−10^ | 4.314×10^−10^ | 6.653×10^−3^ | 5.077×10^−3^ |
|  | 4 cycles | 0.186 | 0.118 | 5.061×10^−10^ | 5.042×10^−10^ | 5.956×10^−3^ | 5.933×10^−3^ |
|  | 5 cycles | 0.124 | 0.127 | 4.119×10^−10^ | 3.785×10^−10^ | 4.847×10^−3^ | 4.454×10^−3^ |
| LH304  (3 months) | 1 cycle | 0.105 | 0.151 | 7.414×10^−10^ | 5.719×10^−10^ | 6.730×10^−3^ | 6.730×10^−3^ |
|  | 2 cycles | 0.117 | 0.141 | 4.401×10^−10^ | 6.452×10^−10^ | 7.592×10^−3^ | 7.592×10^−3^ |
|  | 3 cycles | 0.080 | 0.125 | 2.904×10^−10^ | 2.342×10^−10^ | 2.756×10^−3^ | 2.756×10^−3^ |
|  | 4 cycles | 0.074 | 0.138 | 4.980×10^−10^ | 3.725×10^−10^ | 4.383×10^−3^ | 4.383×10^−3^ |
|  | 5 cycles | 0.093 | 0.115 | 6.050×10^−10^ | 3.467×10^−10^ | 4.080×10^−3^ | 4.080×10^−3^ |
| LH304  (9 months) | 1 cycle | 0.101 | 0.119 | 5.438×10^−10^ | 4.469×10^−10^ | 6.399×10^−3^ | 5.259×10^−3^ |
|  | 2 cycles | 0.093 | 0.118 | 6.952×10^−10^ | 4.684×10^−10^ | 8.180×10^−3^ | 5.513×10^−3^ |
|  | 3 cycles | 0.073 | 0.137 | 7.663×10^−10^ | 6.515×10^−10^ | 9.017×10^−3^ | 7.666×10^−3^ |
|  | 4 cycles | 0.083 | 0.145 | 6.113×10^−10^ | 5.549×10^−10^ | 7.193×10^−3^ | 5.529×10^−3^ |
|  | 5 cycles | 0.075 | 0.137 | 4.100×10^−10^ | 3.876×10^−10^ | 4.824×10^−3^ | 4.561×10^−3^ |

**Supplementary Table 6. *i*_corr_ for the LH304 samples of PDP measurements in the 3.5 wt. % NaCl aqueous solution at different temperature.**

| Temperature | $i_{\mathrm{corr}}$(A·cm^−2^) | |  |
| --- | --- | --- | --- |
|  | Anodic | Cathodic |  |
| 20 | 5.364×10^−10^ | 4.474×10^−10^ |  |
| 30 | 8.790×10^−10^ | 1.135×10^−9^ |  |
| 40 | 1.589×10^−9^ | 3.436×10^−9^ |  |
| 50 | 3.908×10^−9^ | 4.887×10^−9^ |  |
| 60 | 6.353×10^−9^ | 6.668×10^−9^ |  |
